# Molecular profiling of *PYL* gene family in sugar beet (*Beta vulgaris L.*) and *BvPYL*2/3 involved in ABA accumulation confers enhanced resistance to *CLS (Cercospora Leaf Spot*)

**DOI:** 10.3389/fpls.2025.1694558

**Published:** 2025-12-04

**Authors:** Changmei Wu, Chunlei Zhao, Yanli Li, Guangzhou Ding

**Affiliations:** 1 College of Modern Agriculture and Ecological Environment, Heilongjiang University, Harbin, China; 2 Sugar Beet Engineering Research Center of Heilongjiang Province, Harbin, China

**Keywords:** *Beta vulgaris*, Cercospora leaf spot, PYL, ABA signaling, resistance mechanism

## Abstract

*Cercospora leaf* sp*ot* (*CLS*), caused by *Cercospora beticola*, is a major threat to sugar beet (*Beta vulgaris L.*) production globally. While abscisic acid (ABA) signaling mediates plant defense responses, the specific roles of ABA receptors (*PYR/PYL/RCAR*) in sugar beet resistance to *CLS* remain unclear. In this study, *10 BvPYL* genes were identified from the sugar beet genome and functionally characterized. Transcriptome profiling showed that *BvPYL2* displayed a stable constitutive expression profile in resistant and susceptible lines during infection, in contrast to *BvPYL3*, whose expression was marked by a significant decrease over the disease progression. Subcellular localization analysis revealed that both *BvPYL2* and *BvPYL3* localize to the plasma membrane, cytoplasm, and nucleus. Yeast two-hybrid assays demonstrated that *BvPYL2* constitutively interacts with the negative regulator *BvPP2C37*, while *BvPYL3* forms an ABA-dependent complex with *BvPP2C37*. Physiological observations showed that resistant plants achieved ABA-mediated stomatal closure to restrict pathogen entry, whereas susceptible plants maintained prolonged stomatal opening, facilitating infection. These results reveal two distinct regulatory mechanisms: *BvPYL2* functions through constitutive interaction with *BvPP2C37*, and *BvPYL3* operates via ABA-dependent signaling, both contributing to stomatal immunity against *CLS*. This study elucidates novel ABA-mediated defense mechanisms in sugar beet and identifies *BvPYL2* and *BvPYL3* as promising targets for breeding *CLS*-resistant germplasm.

## 1 Introduction

*Beta vulgaris L.*, commonly known as sugar beet, is a biennial root crop belonging to the Amaranthaceae family and ranks among the most economically significant sources of sucrose, contributing approximately 30% of global sugar production (Sun et al., 2024). In addition to its industrial importance, sugar beet possesses notable medicinal value due to its rich content of bioactive compounds, including betacyanins, betaxanthins, and phenolic acids, which exhibit potent antioxidant, anti-inflammatory, and hepatoprotective properties (Stander et al., 2021; Punia Bangar et al., 2022). The biosynthesis and accumulation of these phytochemicals are strongly influenced by environmental growing conditions. However, sugar beet cultivation faces significant challenges, including abiotic stressors such as drought, salinity, and extreme temperatures, as well as biotic threats such as *Cercospora leaf* sp*ot* (*CLS*) (AlKahtani et al., 2021; Alavilli et al., 2023), a severe fungal disease caused by *Cercospora beticola*, which can result in yield losses of 30–50% during severe outbreaks.

Abscisic acid (ABA) is a pivotal phytohormone that regulates a wide range of physiological processes in plants, acting as a signaling molecule with dual roles in developmental regulation and stress response mechanisms. During plant growth and development, ABA plays a crucial role in controlling key developmental transitions, including the maintenance of seed dormancy, suppression of germination, and the initiation of senescence (Zhao et al., 2022). Simultaneously, it functions as a central component of plant defense systems, orchestrating responses to biotic stresses such as pathogen infection, as well as mediating adaptive responses to abiotic stresses including drought, high salinity, and low temperatures (Wei et al., 2025). The pleiotropic effects of ABA signaling underscore its essential role in ensuring plant survival and fitness under varying environmental conditions. As a classical stress-responsive hormone, ABA exhibits dynamic accumulation patterns under adverse environmental stimuli, triggering complex signaling cascades that enhance plant tolerance and survival (Wang et al., 2021). Its physiological regulatory functions are highly context-dependent: under drought conditions, ABA-induced stomatal closure reduces transpirational water loss, thereby preserving cellular water potential and turgor pressure (Liu et al., 2024); during pathogen attack, ABA modulates the expression of defense-related genes through extensive transcriptional reprogramming, thus enhancing immune responses (Hsu et al., 2021). This context-dependent regulatory capability underscores the versatility of ABA as a central coordinator in mediating plant adaptation to environmental stressors.

The activation of ABA signaling is initiated through the specific molecular recognition of abscisic acid (ABA) by dedicated receptor proteins. In plants, the core perception system for ABA involves the Pyrabactin Resistance 1 (*PYR1*)/*PYR1-Like* (*PYL*)/Regulatory Component of ABA Receptor (*RCAR*) family of proteins, commonly known as *PYL* receptors (García-Andrade et al., 2020). These receptors possess a highly conserved START (star-related lipid transfer) domain, which mediates ABA binding and facilitates subsequent signal transduction. Notably, *PYL* proteins exhibit distinct subcellular localization patterns, being present in the cytoplasm, nucleus, and plasma membrane (Wang et al., 2018), thereby enabling comprehensive ABA sensing across cellular compartments and ensuring rapid responses to hormonal signals in diverse cellular environments. Structural analyses have demonstrated that ABA binding induces significant conformational changes in *PYL* receptors (Ali et al., 2020), generating functional interaction surfaces for downstream signaling partners—particularly clade A protein phosphatases 2C (*PP2Cs*). The formation of *PYL-PP2C* complexes represents the key initiating event in the canonical ABA signaling pathway, ultimately leading to the activation of *SnRK2* kinases and the phosphorylation of target proteins involved in both stress responses and developmental regulation. The functional diversity of *PYL* receptors is reflected in their distinct spatiotemporal expression profiles and differential responsiveness to environmental cues. In the model plant *Arabidopsis thaliana*, systematic characterization of its 14 *PYL* members has revealed specialized roles: *AtPYL5* and *AtPYL9* are primarily involved in drought adaptation (Zhao et al., 2017; Quan et al., 2018), *AtPYR1* and *AtPYL4* regulate stomatal dynamics (De Brasi-Velasco et al., 2023), while *AtPYL8* and *AtPYL9* contribute to root architecture modulation under stress conditions (Yang Y. et al., 2024). Comparative studies across crop species further highlight both the conservation and functional specialization of *PYL* homologs in stress adaptation. For instance, *OsPYL3* in rice (*Oryza sativa*) enhances drought tolerance through ABA-mediated stomatal regulation (Lenka et al., 2018); *TaPYL4* in wheat (*Triticum aestivum*) plays a role in cold acclimation and frost resistance (Xue et al., 2021; Liu Y. et al., 2022; Zhang et al., 2022); and *ZmPYL9* in maize (*Zea mays*) promotes salinity tolerance via ABA-dependent transcriptional networks (He et al., 2018). These findings underscore the evolutionary conservation of *PYL*-mediated ABA signaling, while also revealing species-specific adaptations in stress response strategies. The differential expression and functional partitioning of *PYL* isoforms enable plants to precisely modulate ABA signaling in accordance with developmental requirements and environmental challenges.

*PYL* receptors also serve as key regulators of plant immune responses against pathogens through ABA-mediated signaling pathways. In *Solanum lycopersicum*, molecular characterization has demonstrated that *SlPYL3* physically interacts with *SlPP2C4* to establish an ABA-dependent defense mechanism against *Botrytis cinerea* infection. Mechanistically, ABA binding induces *SlPYL3* to suppress *SlPP2C4* phosphatase activity, thereby activating the kinase *SlSnRK2.6.* This kinase subsequently phosphorylates the transcription factor *SlABF2*, leading to the transcriptional activation of pathogenesis-related genes (*SlPR1* and *SlPR5*), which confer resistance to gray mold disease (Chen et al., 2016). Parallel studies on pitaya (*Hylocereus undatus*) have shown that under canker disease stress, the expression of *HuPYL4* and *HuPP2C12* exhibits a coordinated response, and they may be involved in regulating the plant’s disease resistance through the ABA signaling pathway. Transcriptome analysis indicates that changes in the expression of *HuPYL4* can affect the transcriptional level of *HuNCED1*, which in turn may participate in the resistance response to canker disease by regulating endogenous ABA levels (Wang M. et al., 2024). Collectively, these findings indicate that *PYL* receptors function as central nodes in ABA-mediated plant immunity across diverse species; the *PYL-PP2C* interaction constitutes a conserved molecular switch in pathogen defense signaling; and genetic manipulation of *PYL* genes can effectively enhance disease resistance through modulation of the ABA signaling pathway.

The *PYL* receptor family serves as an evolutionarily conserved regulatory hub coordinating phytohormone signaling and stress responses in plants. As a major sucrose-producing crop, sugar beet (*Beta vulgaris L.*) is increasingly affected by production challenges associated with *Cercospora leaf* sp*ot (CLS)*, a disease caused by *Cercospora beticola Sacc*. However, the functional characterization of this gene/protein in this crop remains incomplete, representing a critical knowledge gap that hinders effective management of this yield-limiting disease (Tan et al., 2023). The pathogen employs a complex biphasic infection strategy, initiating with seedborne transmission that frequently involves co-infection with *Phoma betae* (Knight et al., 2020; Rangel et al., 2020). The infection process progresses from initial biotrophic colonization to necrotrophic expansion, severely impairing photosynthetic capacity (Hendershot et al., 2025) and disrupting carbohydrate metabolism (Yang et al., 2025). Although postharvest root characteristics remain largely unaffected (Nelson et al., 2024), infected plants display significantly elevated respiratory activity and increased susceptibility to secondary infections (Hendershot et al., 2025). Sugar beet activates a multi-tiered defense strategy that incorporates both structural barriers, such as cell wall fortification, and biochemical responses, *PYL*-regulated activation of ABA and jasmonate signaling pathways (Yang et al., 2025), and protective interactions with the microbiome (Broccanello et al., 2022). These findings delineate three pivotal research imperatives: mechanistic dissection of *BvPYL*-mediated defense signaling in sugar beet, implementation of integrated pathogen management frameworks synergizing host genetic resistance with microbiome-mediated protection and exploration of novel resistance breeding strategies (Wolfgang et al., 2023; Baryga et al., 2025). Concerted investigation of these priorities will not only address fundamental knowledge deficits in ABA receptor biology but also translate into scalable solutions for sustainable *CLS* mitigation in commercial sugar beet production.

Although abscisic acid (ABA)-mediated defense responses have been associated with resistance to *Cercospora leaf* sp*ot* (*CLS*), the specific role of *PYR/PYL/RCAR (PYL)* receptors in regulating this process in sugar beet (*Beta vulgaris*) remains unclear. Cross-species comparative studies have conclusively demonstrated that *PYL* proteins function as key regulators of ABA sensitivity during pathogen infection, modulating downstream defense signaling pathways in model and crop species such as *Arabidopsis thaliana*, rice, and peanut. However, the molecular mechanisms through which *PYL* receptors regulate *CLS* resistance—particularly how they integrate into ABA signaling to counteract *Cercospora beticola* infection—have not yet been elucidated in sugar beet. To address this knowledge gap, the present study conducted genome-wide identification followed by systematic functional characterization. The experimental approach encompassed profiling of expression dynamics during *CLS* infection progression in both resistant and susceptible materials, validation of critical protein-protein interactions through yeast two-hybrid assays, and correlation of expression patterns with ABA accumulation kinetics. Elucidation of the functional mechanisms underlying ABA-mediated *CLS* resistance provides novel insights into genotype-dependent defense responses in sugar beet, while establishing valuable theoretical frameworks and molecular targets for breeding *CLS*-resistant materials.

## 2 Materials and methods

### 2.1 Plant materials

Leaf samples were collected from sugar beet (*Beta vulgaris L.*) materials exhibiting contrasting resistance levels to *Cercospora leaf* sp*ot* (*CLS*), which were grown in plastic pots within a controlled indoor environment: the susceptible materials KWS6661 and the resistant materials 81GM241. The plants were maintained in a controlled indoor growth facility and selected at a comparable developmental stage—the three-pair true leaf stage—The inoculation procedure was initiated at this growth stage using a *C. beticola* strain isolated from diseased sugar beet leaves. The fungus was propagated on PDA under constant light to induce spore production. The resulting conidia were collected into a sterile aqueous suspension, which was calibrated to 1×10^6^ spores/mL. Each pot received a 10 mL application of this inoculum through foliar spraying, with control pots receiving 10 mL of sterile water. The strain’s virulence had been pre-confirmed in pathogenicity assays, with challenged susceptible plants displaying typical *CLS* symptoms. Disease progression was categorized into four distinct stages: asymptomatic (control), early infection (5–10% leaf coverage with discrete lesions ≤2 mm in diameter), moderate infection (25–35% coverage with coalescing lesions), and advanced infection (65–75% coverage with extensive necrosis) (Schmittgen, 2015; Jay et al., 2020). For each disease stage, three biological replicates (comprising nine plants per materials) were collected. To maintain RNA stability, collected samples were immediately snap-frozen in liquid nitrogen and subsequently kept at –80°C until further processing for RNA isolation. For representative sampling, leaves were harvested from the middle canopy layer (3rd to 5th leaf positions) between 9:00 and 11:00 AM to reduce potential diurnal effects. The sampling environment was controlled at 25–28°C and 70–85% relative humidity. All plants were grown under uniform agronomic management in an indoor growth facility to ensure consistent environmental conditions.

### 2.2 Transcriptome and gene expression analysis

Transcriptome and gene expression analyses were performed on leaf samples from two sugar beet (*Beta vulgaris L.*) materials with differential *Cercospora leaf* sp*ot* resistance: 81GM241 (resistant) and KWS6661 (susceptible). Plants were sampled at the three-pair true leaf stage to ensure developmental uniformity. A total of nine plants per materials were divided into three biological replicates, representing control, early infection, and disease onset groups (three biological replicates per group, yielding 24 samples in total). Following RNA extraction and library preparation, Illumina sequencing was performed by Majorbio Biotechnology Co., Ltd. (Shanghai). Raw sequencing reads were processed using a standardized bioinformatics pipeline on the Majorbio Cloud Platform (https://cloud.majorbio.com) (Han et al., 2024): Fastp 0.23.2 was employed for quality assessment, SeqPrep 1.3.2 for adapter trimming, and Sickle 1.33 for filtering low-quality reads, sequences containing ambiguous bases, and short fragments (M et al., 2016; Bolger et al., 2014). High-quality clean reads were mapped to the sugar beet reference genome (Ref-Beet-1.2) with RSEM 1.3.3 to quantify transcript abundance in FPKM (fragments per kilobase of transcript per million mapped reads). Differentially expressed genes (DEGs) were identified using DESeq2 1.38.3 with strict statistical thresholds. Functional interpretation of the DEGs was conducted through Gene Ontology (GO) enrichment analysis, covering biological processes, molecular functions, and cellular components, along with Kyoto Encyclopedia of Genes and Genomes (KEGG) pathway analysis to elucidate metabolic and signaling pathways related to *Cercospora leaf spot* (*CLS*) resistance (Kanehisa and Goto, 2000). Analyses were conducted using the R package clusterProfiler 4.0 (Chen C. et al., 2020), and results were visualized as hierarchical clustering heatmaps using TBtools 2.0 (https://github.com/CJ-Chen/TBtools/releases) (Chen C. et al., 2020), enabling comparative analysis of transcriptional profiles between resistant and susceptible materials across infection stages. The data supporting the findings of this study have been deposited in the National Center for Biotechnology Information (NCBI) repository under the BioProject accession number PRJNA1288145 and are accessible through the following URL: https://submit.ncbi.nlm.nih.gov/subs/, PRJNA1288145.

### 2.3 Genome-wide identification and evolutionary analysis of BvPYL family genes

The protein sequences of *Beta vulgaris* were obtained from the Sugar Beet Genome Resource (https://bvseq.boku.ac.at/, accessed). The HMM profile (PF10604) corresponding to the conserved *PYL* domain was downloaded from the PFAM database (http://pfam.xfam.org/) and used to search the *Beta vulgaris* proteome using HMMER (Yang J. et al., 2024). Candidate *BvPYL* genes were further validated by analyzing their domain architectures through the NCBI Conserved Domains Database (CDD) (https://www.ncbi.nlm.nih.gov/Structure/cdd/) and SMART (http://smart.embl-heidelberg.de/, accessed on 22 October 2024). For promoter analysis, 3000-bp upstream regions of *BvPYL* genes were scanned for potential cis-acting regulatory elements using PlantCARE (http://bioinformatics.psb.ugent.be/webtools/plantcare/html/). Chromosomal localization of *BvPYL* genes was determined using MG2C (http://mg2c.iask.in/mg2c_v2.0/). To investigate evolutionary relationships, a phylogenetic tree was constructed using MEGA 7.0 with the neighbor-joining method (1000 bootstrap replicates) (Tree visualization by one table (tvBOT): A web application for visualizing, modifying and annotating phylogenetic trees | Nucleic acids research). This analysis involved aligning *BvPYL* sequences with orthologous sequences from *Arabidopsis thaliana* (TAIR, https://www.arabidopsis.org/, accessed on 2 November 2024) and *Oryza sativa* (Ensembl Plants, https://ensemblgenomes.org/plants/). Additionally, collinearity analysis was conducted using MCScanX to detect intragenomic duplication events within *Beta vulgaris* and to assess interspecific syntenic relationships with *Arabidopsis thaliana* and *Oryza sativa* (Wang Y. et al., 2024).

### 2.4 Structural and functional characterization of BvPYL genes

The exon-intron organization of *BvPYL* genes was analyzed by aligning coding sequences with their corresponding genomic sequences using the Gene Structure Display Server (Hu et al., 2015) (GSDS; http://gsds.cbi.pku.edu.cn/). The resulting gene structures were subsequently visualized using TBtools (https://github.com/CJ-Chen/TBtools/releases). For functional characterization, conserved protein motifs were identified using the MEME Suite (https://meme-suite.org/meme/) with the following parameters: a maximum of 15 motifs and a minimum motif width of 6 amino acids (Nystrom and McKay, 2021). The distribution patterns and structural organization of these motifs were further examined to evaluate potential functional conservation among *BvPYL* family members. Additionally, all identified protein sequences were analyzed for their physicochemical properties, including molecular weight, theoretical isoelectric point (pI), and grand average of hydropathicity (GRAVY), using the ProtParam tool on the ExPASy platform (Duvaud et al., 2021) (http://web.expasy.org/protparam/).

### 2.5 Quantitative reverse transcription-PCR analysis of BvPYL genes

To investigate the dynamic expression patterns of *BvPYL* genes in response to *Cercospora* infection, qRT-PCR analysis was performed on leaf samples collected at different time points post-inoculation. Total RNA was isolated from 100 mg of sugar beet leaf tissue ground in liquid nitrogen with the TaKaRa MiniBEST Universal RNA Extraction Kit (Takara Bio, Japan), incorporating an on-column DNase I treatment to remove genomic DNA. RNA quality was assessed through agarose gel electrophoresis and verified by an RNA Integrity Number (RIN) exceeding 8.0. Quantification of RNA was conducted with a NanoDrop 2000 spectrophotometer (Thermo Fisher Scientific). First-strand cDNA was generated from 1 μg of total RNA via PrimeScript™ II Reverse Transcriptase (Takara Bio) and oligo(dT) primers. Gene-specific primers targeting *BvPYL* genes and the reference gene *BvACTIN* were designed with Primer3Plus to produce amplicons ranging from 100 to 300 bp (primer sequences provided in **Supplementary Table 1**). Quantitative real-time PCR (qRT-PCR) was performed on a QuantStudio 5 Real-Time PCR System (Applied Biosystems) with TB Green™ Premix Ex Taq™ II (Takara Bio) under the following protocol: initial denaturation at 95°C for 30 s, 40 cycles of 95°C for 5 s and 60°C for 30 s. Relative expression was determined using the 2^−ΔΔCt method, with normalization to *BvACTIN* and calibration against the 0 h post-treatment sample. The experiment included three biological replicates per sample, each run in two technical replicates. Data analysis and visualization were performed using GraphPad Prism version 9.0.0.

### 2.6 Subcellular localization analysis of BvPYL2 and BvPYL3

Initial subcellular localization predictions for *BvPYL2* and *BvPYL3* were conducted using the CellPLoc server (http://www.csbio.sjtu.edu.cn/bioinf/Cell-PLoc/). The full-length open reading frames of both genes were amplified by PCR using gene-specific primers (primer sequences are listed in **Supplementary Table 1**), which were designed to include 5’ homologous arms complementary to the pHK-35S-GLosGFP expression vector (Boyuan Biotech, China) for seamless cloning. This vector contains a CaMV 35S promoter and a plant codon-optimized GFP reporter gene separated by a flexible linker. PCR amplification was carried out using 2×BioRun Pfu PCR Mix under the following thermal cycling conditions: initial denaturation at 98°C for 3 min; 25–30 cycles of 98°C for 15 s, 55–60°C for 15 s, and 72°C for 30–60 s (with an extension rate of 1 kb/min); followed by a final extension at 72°C for 5 min. Purified PCR products were cloned into the linearized vector using 2×Seamless Cloning Mix at 50°C for 30–60 min, resulting in the generation of pHK-35S-*BvPYL2-GLos*GFP and pHK-35S-*BvPYL3*-GLosGFP constructs. These recombinant plasmids, along with a 35S: GFP control vector, were transiently expressed in tobacco leaves via Agrobacterium-mediated transformation. Subcellular localization was analyzed 48 h post-infiltration using a Zeiss LSM780 confocal laser scanning microscope following dark incubation.

### 2.7 Yeast two-hybrid analysis of BvPYL2/BvPYL3 and BvPP2C37 interactions

Protein-protein interactions between *BvPYL2*, *BvPYL3* and *BvPP2C37* were examined using the Matchmaker Gold Yeast Two-Hybrid System (Clontech, USA). The coding sequences of *BvPYL2* and *BvPYL3* were cloned into the pGADT7 prey vector, while *BvPP2C37* was inserted into the pGBKT7 bait vector (Primer sequences are provided in **Supplementary Table 1**). Following co-transformation into Y2HGold competent cells, transformants were first selected on SD/-Leu/-Trp medium. For interaction testing, positive colonies were resuspended to OD_600_ = 0.2 and serially diluted (10-fold dilutions to 0.02, 0.002, and 0.0002). Aliquots (10 μL) were spotted onto: SD/-Leu/-Trp control medium and stringent selection medium (SD/-Leu/-Trp/-His/-Ade) containing X-α-Gal and aureobasidin A. Plates were incubated at 30°C for 4–5 days before evaluating interactions based on colony growth and color development.

### 2.8 ABA quantification in sugar beet leaves via HPLC-MS/MS

Leaf samples from resistant (R) and susceptible (S) sugar beet (*Beta vulgaris*) genotypes were collected based on a timeline defined from the three-pair true leaf stage: at 12 days (designated 0 days post-inoculation, 0 dpi), 24 days (12 dpi), and 48 days (36 dpi) from this starting point, with three biological replicates per genotype. Upon collection, samples were immediately flash-frozen in liquid nitrogen and maintained at −80°C until further processing. For ABA extraction, 0.2 g of frozen tissue was homogenized and incubated overnight at 4°C in 5.0 mL of chilled acetonitrile spiked with 30 μL of deuterated ABA (d6-ABA) as an internal standard. After centrifugation (12,000 × g, 10 min, 4°C), the supernatant was recovered and the residue subjected to a second extraction using 3.0 mL of acetonitrile. The pooled extracts were cleaned with 200 mg of CNW C18 QuEChERS sorbent, dried under a nitrogen stream, and redissolved in 150 μL of methanol. The reconstituted samples were filtered through a 0.22 μm membrane before HPLC-MS/MS analysis. Separation was carried out on a Waters XSelect^®^ HSS T3 column (2.1 × 150 mm, 2.5 μm) with a gradient program (mobile phase A: 0.1% formic acid in water; B: acetonitrile) at 0.3 mL/min (Herrera et al., 2010; Huang et al., 2014). ABA was quantified in multiple reaction monitoring (MRM) mode, with compound identification verified by retention time and specific ion transitions relative to authentic standards. A seven-point calibration curve (r² > 0.99) constructed from ABA standards was used for quantification, with results normalized to the internal standard. To analyze the temporal dynamics of ABA, the quantified ABA concentrations were converted to Relative Quantification (RQ) values. The RQ value for each sample was calculated by dividing its ABA concentration by the mean ABA concentration of the resistant genotype at the 0dpi time point, establishing this reference value as the baseline (RQ = 1). Statistical significance was assessed by one-way ANOVA followed by Tukey’s test, considering p < 0.05 as significant.

### 2.9 Stomatal observation of sugar beet leaf surface

Stomatal dynamics were examined in sugar beet (*Beta vulgaris*). Daily leaf sampling was carried out over a 15-day period to monitor stomatal responses during disease development, with asymptomatic leaves from the same plants as controls. For each time point, three biological replicates were analyzed. Leaf segments (1 cm²) were carefully excised while avoiding major veins, then immediately observed under an optical microscope.

## 3 Results

### 3.1 Modular organization of ABA signaling networks in sugar beet during pathogen infection

**Figure 1:**
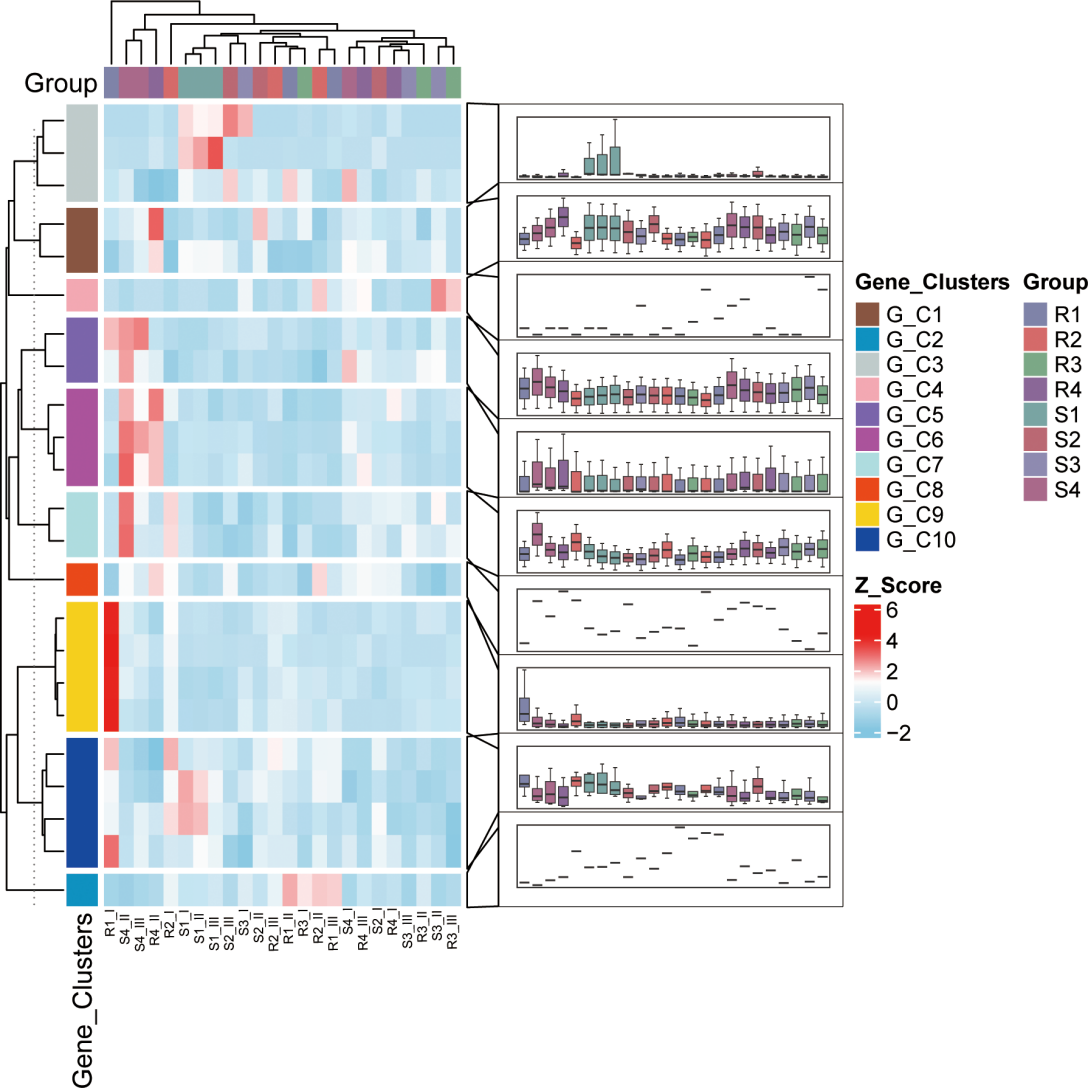
Co-expression clustering analysis of ABA signaling-related genes in sugar beet during *Cercospora beticola* infection. The heatmap in the left area displays gene expression patterns across a single resistant and a single susceptible sugar beet genotype at four distinct disease progression stages (designated R1-R4 for the resistant genotype and S1-S4 for the susceptible genotype, corresponding to asymptomatic, early, moderate, and advanced infection stages as defined in the Methods), where each column corresponds to a sample, each row to a gene, and expression levels normalized by Z - score are represented by color intensity with red signifying high expression and blue signifying low expression. The sample clustering dendrogram in the top - left portion categorizes samples based on the similarity of their gene expression profiles. The cluster assignment via right – hand colored blocks uses different colors to represent ten distinct co - expression modules, namely G_C1 to G_C10. The box plots in the right area depict the expression distribution patterns for each cluster, with the Y-axis showing Z-score normalized expression values, the center line indicating the median, the box showing the interquartile range, and the whiskers illustrating the data distribution, and this analysis reveals distinct ABA signaling modules associated with disease resistance and susceptibility.

Transcriptional dynamics of co-expressed gene modules (G_C1-G_C10) in sugar beet (*Beta vulgaris*) during *Cercospora beticola* infection were analyzed through integrated co-expression networks using hierarchical clustering and Z-score normalization. The analysis identified ten functionally distinct gene clusters exhibiting differential expression patterns across four distinct disease progression stages (as defined in the Methods) in a single resistant genotype (designated R1-R4, corresponding to asymptomatic, early, moderate, and advanced infection stages) and a single susceptible genotype (designated S1-S4, representing the same respective stages) (**Figure 1**). Resistance-associated clusters, particularly G_C1, demonstrated significant upregulation (Z-score >4) in resistant lines, with functional annotation revealing enrichment of positive regulators including *SnRK2* kinases, suggesting their potential role in immune responses. Susceptibility-associated clusters such as G_C6 showed preferential activation in susceptible genotypes and contained negative regulators including *PP2C* phosphatases, potentially indicative of suppressed defense signaling. While these modules contained ABA signaling components (*PYL-PP2C-SnRK2*), their precise functional relationship with disease outcomes requires further experimental validation. Intermediate clusters including G_C3 displayed dynamic expression patterns during resistance-susceptibility transitions, potentially functioning as regulatory nodes that integrate membrane-based signal perception with nuclear transcriptional responses (**Supplementary Table S2**). This modular organization of stress-responsive gene networks provides a framework for understanding disease outcome determination, where specific cluster activation patterns correlate with resistance or susceptibility phenotypes. The observed differential regulation of positive and negative regulatory components suggests a potential role for ABA-related signaling pathways in pathogen response, though mechanistic validation of these relationships remains an important direction for future research.

### 3.2 Functional enrichment analyses of BvPYL genes in ABA-mediated defense

**Figure 2:**
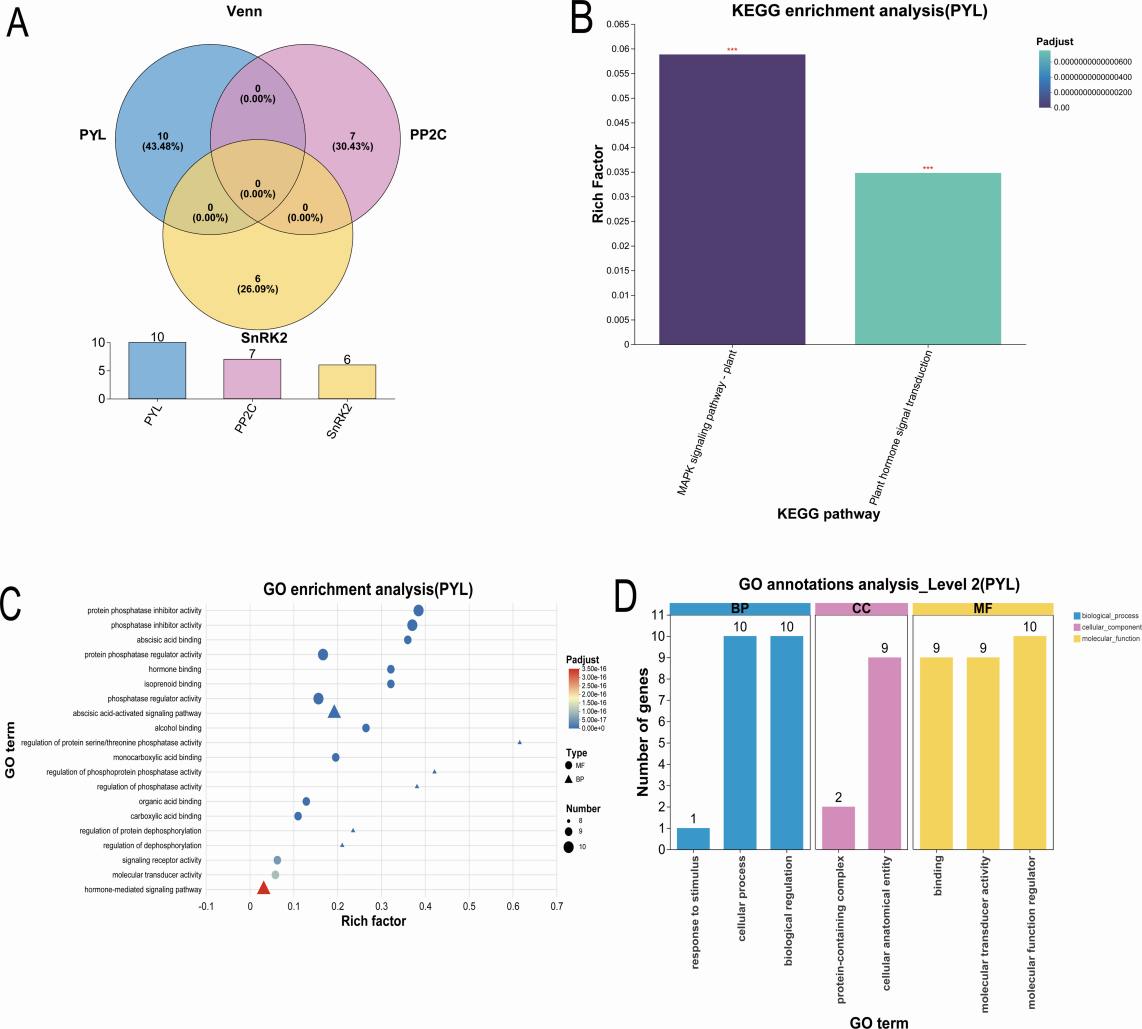
Functional enrichment analyses of *BvPYL* genes in ABA - mediated defense responses. **(A)** Venn analysis of core ABA signaling components. **(B)** KEGG enrichment analysis showing significant involvement of *BvPYL*-associated genes in plant MAPK signaling and hormone transduction pathways (P < 0.001). **(C)** Gene Ontology (GO) enrichment of *BvPYL*-induced differentially expressed genes (DEGs), highlighting molecular functions and biological processes. **(D)** Level 2 GO categorization of *BvPYL* genes, emphasizing their roles in cellular compartments, signal transduction, and binding activities.

Comprehensive functional analyses revealed distinct regulatory mechanisms of *BvPYL* genes in sugar beet’s ABA-mediated resistance to *CLS*. Venn analysis showed complete genetic separation between core ABA signaling components (*PYL, PP2C, and SnRK2* families) (**Figure 2A**), confirming their interaction occurs through protein-level crosstalk rather than shared genetic elements. This aligns with the well-established ABA signaling paradigm: ABA binding to *PYL* receptors relieves *PP2C*-mediated inhibition of *SnRK2* kinases, thereby activating downstream stress responses. KEGG pathway analysis revealed significant enrichment (P < 0.001) of *BvPYL*-associated genes in the plant MAPK signaling and hormone transduction pathways, suggesting their potential involvement in stress adaptation responses. (**Figure 2B**) (Supplementary material: **Supplementary Table S3**). The molecular function profile of *BvPYL* genes showed remarkable specialization in ABA signaling components. GO enrichment identified association with protein phosphatase inhibitor activity (Rich factor >5) and abscisic acid binding (Rich factor >4) (**Figure 2C**) (Supplementary material: **Supplementary Table S4**), highlighting their dual role as ABA receptors and phosphatase regulators. Biological process annotation revealed predominant involvement in ABA-activated signaling pathways and cellular stress responses. These findings establish *BvPYL* genes as central players in both ABA perception and downstream signal transduction during pathogen defense. Cellular characterization through Level 2 GO analysis provided further functional insights (**Figure 2D**). *BvPYL*-associated genes predominantly localized to general cellular compartments rather than specialized protein complexes, suggesting broad regulatory functions. Molecular annotation showed equal distribution between signal transduction and binding activities, consistent with their roles as receptors and signaling hubs. Notably, the limited representation in “response to stimulus” categories suggests *BvPYL* genes primarily function in signal integration rather than initial stress perception.

Collectively, these analyses are consistent with a model in which *BvPYL* genes contribute to multi-layered defense responses, potentially through roles in ABA perception and binding, phosphatase regulation, hormone signaling modulation, and cellular stress response coordination. The integrated enrichment profile further implies that *BvPYL* genes could act as regulatory nodes, possibly facilitating crosstalk between ABA signaling and pathogen defense across molecular, cellular, and physiological levels. Together, these findings propose a working model for *CLS* resistance mechanisms in sugar beet, while recognizing that definitive functional relationships will require further experimental validation.

### 3.3 Genome-wide identification and characterization of the BvPYL gene family in *Beta vulgaris*

A genome-wide identification and multi-dimensional characterization of the abscisic acid (ABA) receptor *PYL (PYR/PYL/RCAR)* gene family was performed in sugar beet (*Beta vulgaris*), including analyses of chromosomal distribution, physicochemical properties, gene structure, conserved motifs, protein sequence alignment, and cis-acting regulatory elements. Genome-wide screening identified 10 *BvPYL* genes unevenly distributed across 18 *Beta vulgaris* chromosomes (Supplementary Material: **Supplementary Figure 1**). Designated *BvPYL1-BvPYL10* according to chromosomal position, these genes exhibited an asymmetric distribution pattern, with most chromosomes containing a single *BvPYL* gene except chromosome 5, which harbored two genes (*BvPYL5* and *BvPYL6*). Analysis of encoded *BvPYL* proteins using ExPASy ProtParam revealed length variations from 151 (*BvPYL7*) to 261 amino acids (*BvPYL10*), molecular weights ranging from 17.2 to 29.5 kDa, and isoelectric points (pI) between 4.47 and 6.70 (**Supplementary Material**: **Supplementary Table S5**).

**Figure 3:**
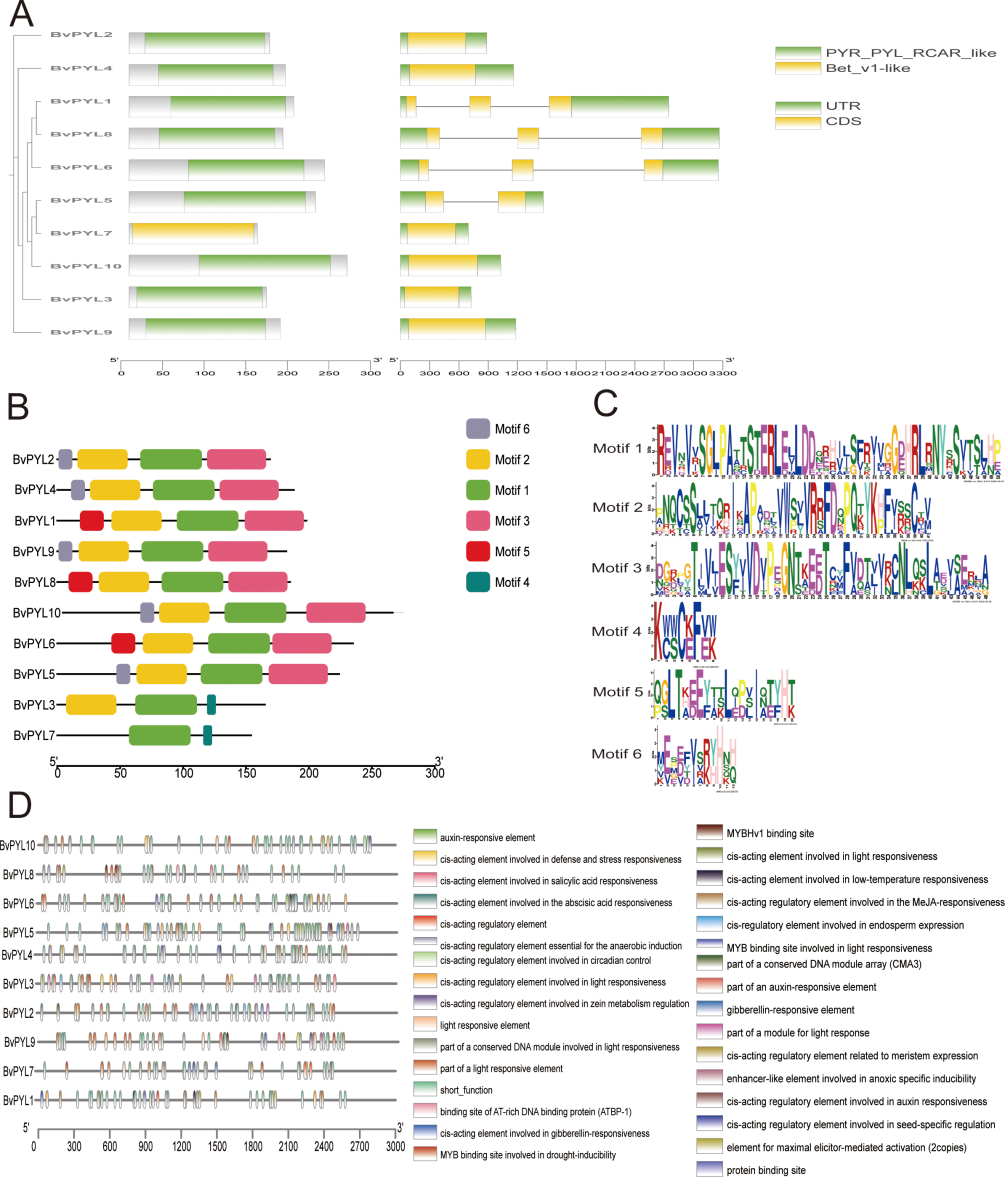
Genome-wide characterization of the *BvPYL* gene family in *Beta vulgaris*. **(A)** Phylogenetic tree of *BvPYL* proteins, integrated with gene structure visualization and subfamily classification. **(B)** Distribution of six conserved motifs across *BvPYL* proteins, with motif positions mapped to protein sequence lengths. **(C)** Sequence logos of the six conserved motifs, where the height of amino acid residues reflects conservation strength. **(D)** Cis-acting regulatory elements in the 3-kb upstream promoter regions of *BvPYL* genes, categorized by functional annotation and color-coded as per the legend.

Structural annotation demonstrated that most *BvPYL* genes contain two exons (**Figure 3A**), indicating conserved gene architecture. MEME-based motif analysis identified six conserved motifs in *BvPYL* proteins (**Figures 3B, C**), likely maintaining structural integrity and functionality. Multiple sequence alignment revealed that all *BvPYL* proteins contain the conserved START-like domain characteristic of the *PYR/PYL/RCAR* family, which is essential for ABA perception and *PP2C* interaction (**Supplementary Material**: **Supplementary Figure 2**) (Insights into the PYR/PYL/RCAR Gene Family in Pomegranates (Punica Granatum L.): A Genome-Wide Study on Identification, Evolution, and Expression Analysis). The strict conservation of this domain across all 10 *BvPYL* proteins supports their functional homology as ABA receptors. Analysis of 3000-bp up stream promoter regions using Plant CARE identified heterogeneous distribution of diverse cis-elements among *BvPYL* paralogs (**Figure 3D**). These elements were categorized into three functional groups: phytohormone-responsive elements (ABA, JA, GA, SA, auxin), stress-responsive elements (anoxia, low temperature, drought, wounding, defense), and elements regulating light responsiveness, metabolism, and meristem-specific expression. ABA-responsive elements showed the highest abundance, while anoxia-specific elements were rare, indicating that *BvPYL* genes are integrated into a complex regulatory network with predominant ABA-mediated control.

### 3.4 Evolutionary dynamics and functional diversification of PYL gene family in sugar beet (*Beta vulgaris*)

**Figure 4:**
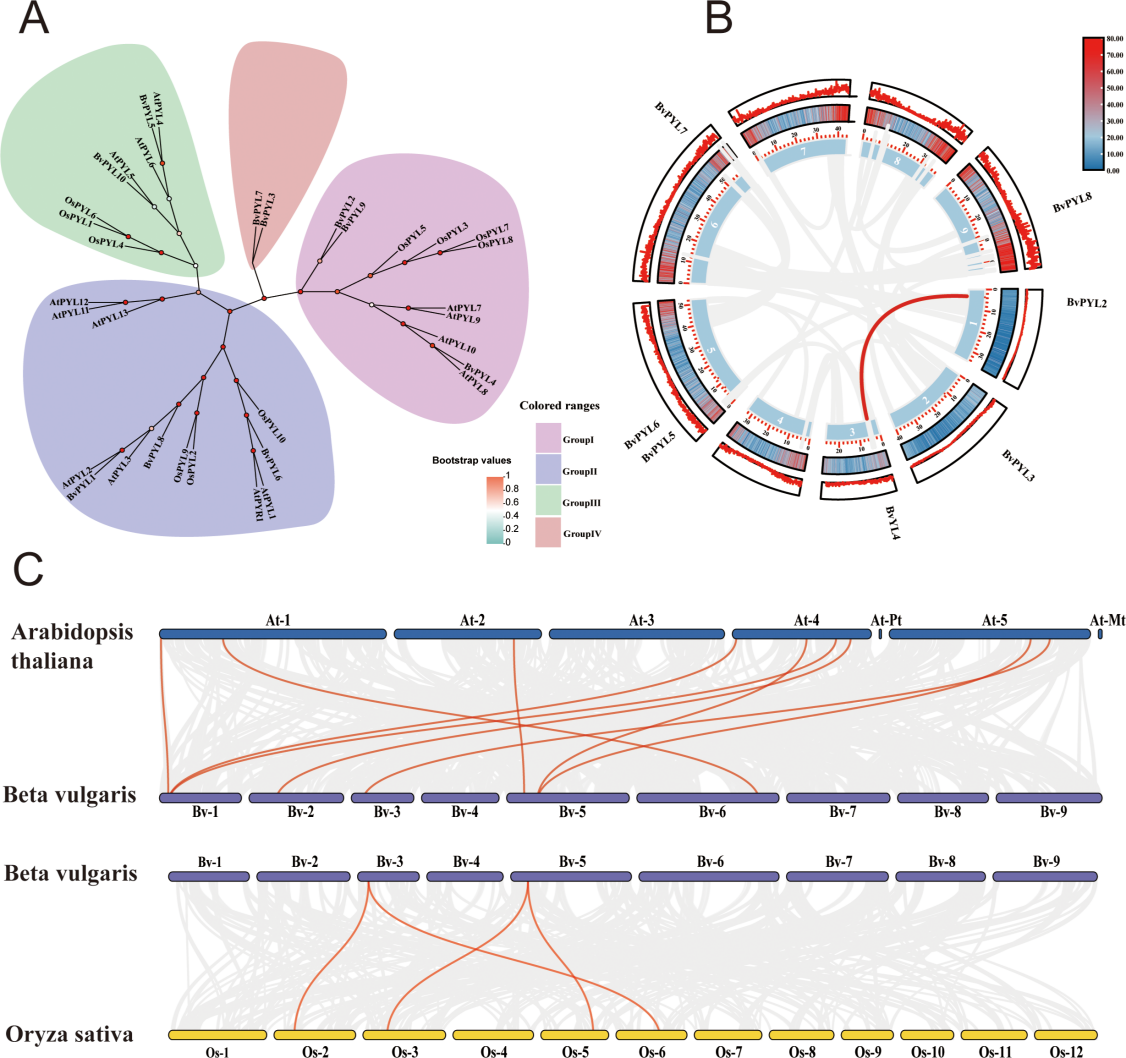
Evolutionary and collinearity analysis of *PYL* genes in sugar beet and model plants. **(A)** Phylogenetic analysis of *PYL* genes from *Beta vulgaris, Arabidopsis thaliana*, and *Oryza sativa*. The neighbor-joining tree was constructed using MEGA 11 (bootstrap value = 1000), with colored ranges denoting four distinct clades (Group I–IV). **(B)** Intraspecific collinearity analysis of *BvPYL* genes in *Beta vulgaris*. Syntenic relationships are indicated by lines, with dark purple lines highlighting connections involving *BvPYL* genes and light purple lines representing other genomic syntenic blocks. **(C)** Interspecific collinearity analysis of *PYL* genes among *B. vulgaris, A. thaliana*, and *O. sativa*. Syntenic relationships are indicated by lines, with dark colors (dark green for *Bv-At*, dark blue for *Bv-Os*) highlighting connections involving *PYL* genes and light colors (light green for *Bv-At*, light blue for *Bv-Os*) representing other genomic syntenic blocks.

To investigate the evolutionary relationships within the *PYL* (Pyrabactin Resistance-Like) gene family in sugar beet (*Beta vulgaris*), we performed a comprehensive phylogenetic analysis using full-length amino acid sequences from *Beta vulgaris*, *Arabidopsis thaliana*, and *Oryza sativa* (**Supplementary Material**: **Supplementary Table S6**).These *PYL* proteins serve as fundamental components of the abscisic acid (ABA) signaling pathway, mediating plant responses to diverse environmental stresses, particularly drought, salinity stress, and pathogen infection (Fidler et al., 2022). Phylogenetic reconstruction using the neighbor-joining method with 1000 bootstrap replicates identified four well-supported clades (I-IV; **Figure 4A**), demonstrating both evolutionary conservation and species-specific divergence patterns among *PYL* genes across the studied species. Clade I comprised 11 members, including *Arabidopsis AtPYL7–10* and sugar beet *(Beta vulgaris) BvPYL2/4/9*. Significantly, *Arabidopsis AtPYL9* is known to mediate drought, high salinity, and osmotic stress responses through ABA signaling pathways, suggesting potential functional conservation in the sugar beet orthologs *BvPYL2/4/9* (Wang et al., 2023). Clade II contained 13 members, featuring Arabidopsis *AtPYR1 (AtPYL1), AtPYL2-3, AtPYL11-13*, along with sugar beet *BvPYL1/6/8*. The inclusion of *Arabidopsis AtPYR1* and *AtPYL2* - established regulators of ABA-dependent stomatal closure and fungal pathogen resistance strongly indicates that the corresponding sugar beet *BvPYL* genes may maintain similar defensive roles against biotic stresses (Dittrich et al., 2019). Clade III, consisting of 8 members, included *Arabidopsis AtPYL4–6* and sugar beet *BvPYL5/10*. Given the well-documented association between *Arabidopsis AtPYL4–6* and drought resistance phenotypes, these findings strongly suggest that *BvPYL5* and *BvPYL10* likely preserve comparable functions in ABA-mediated drought response mechanisms (Shukla et al., 2019). Clade IV emerged as a distinct evolutionary lineage exclusively containing two sugar beet-specific genes (*BvPYL3* and *BvPYL7*). Their unique phylogenetic distribution, with no detectable orthologs in other species, implies that sugar beet may have evolved specialized adaptive mechanisms through these genes, potentially enabling responses to species-specific biotic or abiotic stress factors. These evolutionary patterns reveal that the sugar beet *PYL* gene family has maintained conserved ABA signaling functions while undergoing lineage-specific diversification. The robust clustering of *BvPYL* genes with functionally characterized *Arabidopsis* and rice orthologs enables reliable prediction of their stress response specializations, particularly regarding:Abiotic stress adaptation (drought and cold tolerance);Biotic stress resistance (pathogen defense mechanisms); This phylogenetic framework provides critical insights for future functional characterization studies aimed at elucidating the specific roles of *BvPYL* genes in enhancing stress resilience in sugar beet.

Gene duplication represents a fundamental mechanism for generating genetic novelty and functional diversification. To elucidate the evolutionary trajectory of *BvPYL* genes in sugar beet (*Beta vulgaris*), comprehensive intra- and interspecific analyses were conducted, integrating whole-genome duplication and collinearity assessments. Intraspecific duplication analysis revealed a single syntenic gene pair (*BvPYL2/BvPYL4*) (**Figure 4B**), indicating segmental duplication likely drove *BvPYL* family expansion; the absence of detectable collinearity among other *BvPYL* genes suggests alternative pathways may have shaped their genomic architecture. Interspecific collinearity analysis with *Arabidopsis thaliana* (eudicot) and *Oryza sativa* (monocot) (**Figure 4C**) identified nine conserved gene pairs between sugar beet and *A. thaliana*, and four between sugar beet and rice. The higher number of eudicot-eudicot (sugar beet–*A. thaliana*) versus eudicot-monocot (sugar beet–rice) collinear pairs reflects phylogenetic divergence, while multiple *BvPYL* genes maintaining collinear relationships with orthologs in both species imply conserved roles as core components of the abscisic acid (ABA) signaling pathway. These integrated analyses elucidate evolutionary conservation within the *PYL* gene family, establish a framework for predicting gene functions via orthologous relationships, and identify lineage-specific *BvPYL* genes potentially underlying unique stress adaptations in sugar beet. Collectively, the findings advance understanding of ABA receptor evolution and provide a foundation for future functional characterization in this crop species.

### 3.5 Expression profiling of BvPYL genes in leaf tissues in response to Cercospora leaf spot

**Figure 5:**
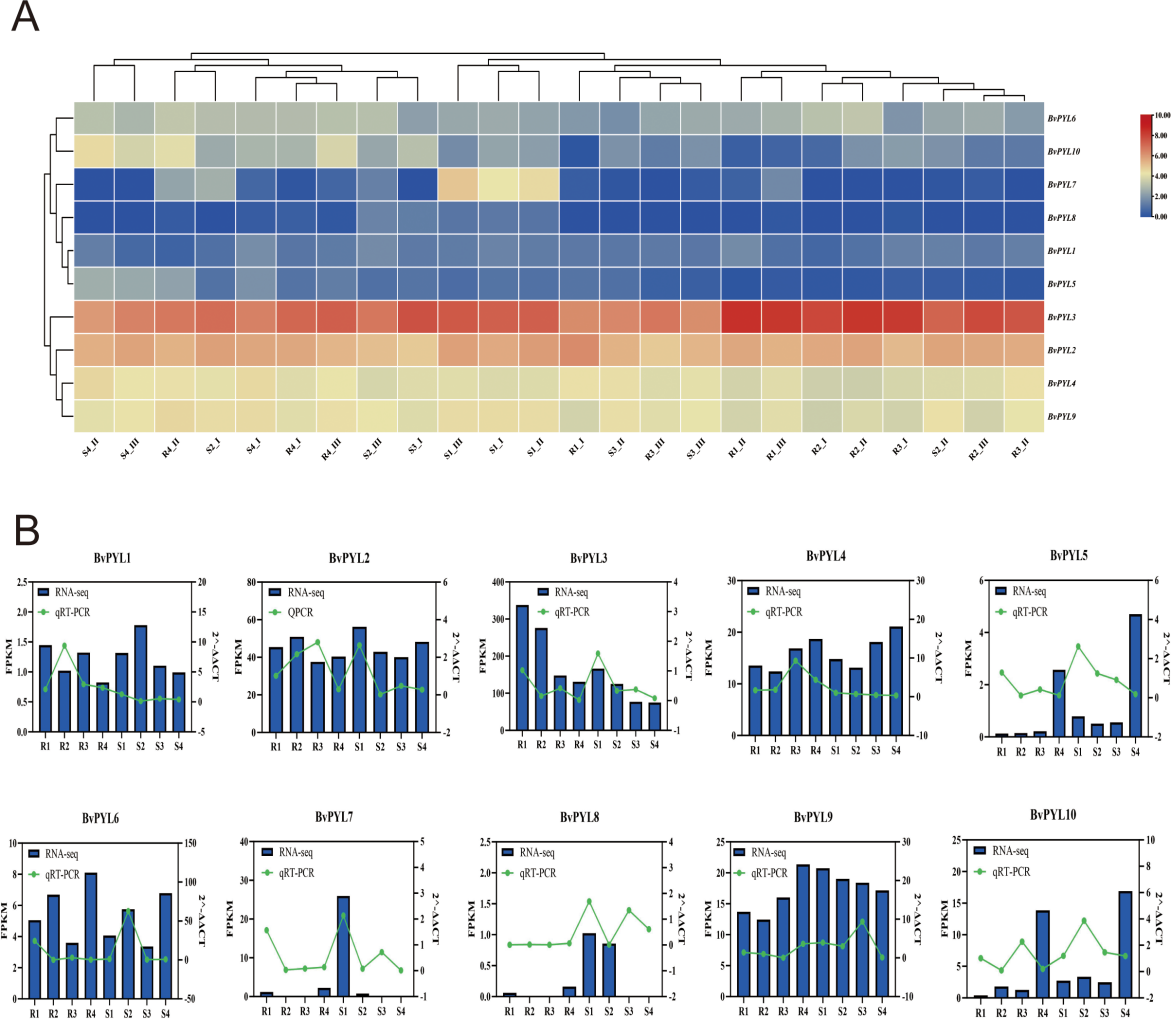
Expression analysis of *BvPYL* genes in sugar beet leaves in response to *Cercospora beticola* infection. **(A)** Heatmap of RNA-Seq expression profiles (FPKM) for the ten *BvPYL* genes at different infection stages (designated R1–R4 for the resistant genotype and S1–S4 for the susceptible genotype, corresponding to asymptomatic, early, moderate, and advanced infection stages as defined in the Methods). **(B)** RNA-Seq (blue bars, left axis: FPKM) and qRT-PCR (green lines, right axis: relative expression) expression profiles for the ten *BvPYL* genes across infection stages (R1–R4 and S1–S4).

High-throughput RNA-seq analysis of sugar beet leaf tissues, the primary infection site of *CLS*, revealed distinct expression patterns among *BvPYL* family members during *CLS* infection (**Supplementary Material**: **Supplementary Table S7**; **Figure 5A**). Transcriptome data showed that primarily *BvPYL2* and *BvPYL3* were abundantly expressed in leaf tissues, positioning them as the primary candidates for mediating ABA signaling during this specific host-pathogen interaction. Four members (*BvPYL4*, *BvPYL6*, *BvPYL9*, and *BvPYL10*) were expressed at low levels (FPKM generally <1), making it difficult to correlate their expression patterns with *CLS* infection, while the remaining four genes (*BvPYL1*, *BvPYL5*, *BvPYL7*, and *BvPYL8*) showed no or negligible expression (FPKM <0.5) in leaf tissues at all time points, suggesting they may function primarily in other organs or under different physiological conditions.

qRT-PCR validation using gene-specific primers (**Supplementary Table 1**) confirmed the RNA-seq data reliability and further elucidated the expression dynamics of *BvPYL2* and *BvPYL3* (**Figure 5B**): *BvPYL2* expression peaked significantly earlier in resistant materials than in susceptible ones, while *BvPYL3* accumulated 2.3-fold higher in resistant varieties. These results strongly suggest *BvPYL2* and *BvPYL3* serve as key regulatory nodes in *CLS* resistance in leaf tissues, with their expression kinetics and magnitude directly correlating with materials resistance levels. Protein interaction predictions further indicate these gene products may form complexes with *PP2C* phosphatases and *SnRK2* kinases, collectively regulating downstream defense genes in the ABA signaling pathway.

### 3.6 Subcellular localization and functional implications of BvPYL2 and BvPYL3 in sugar beet defense against *Cercospora beticola*

**Figure 6:**
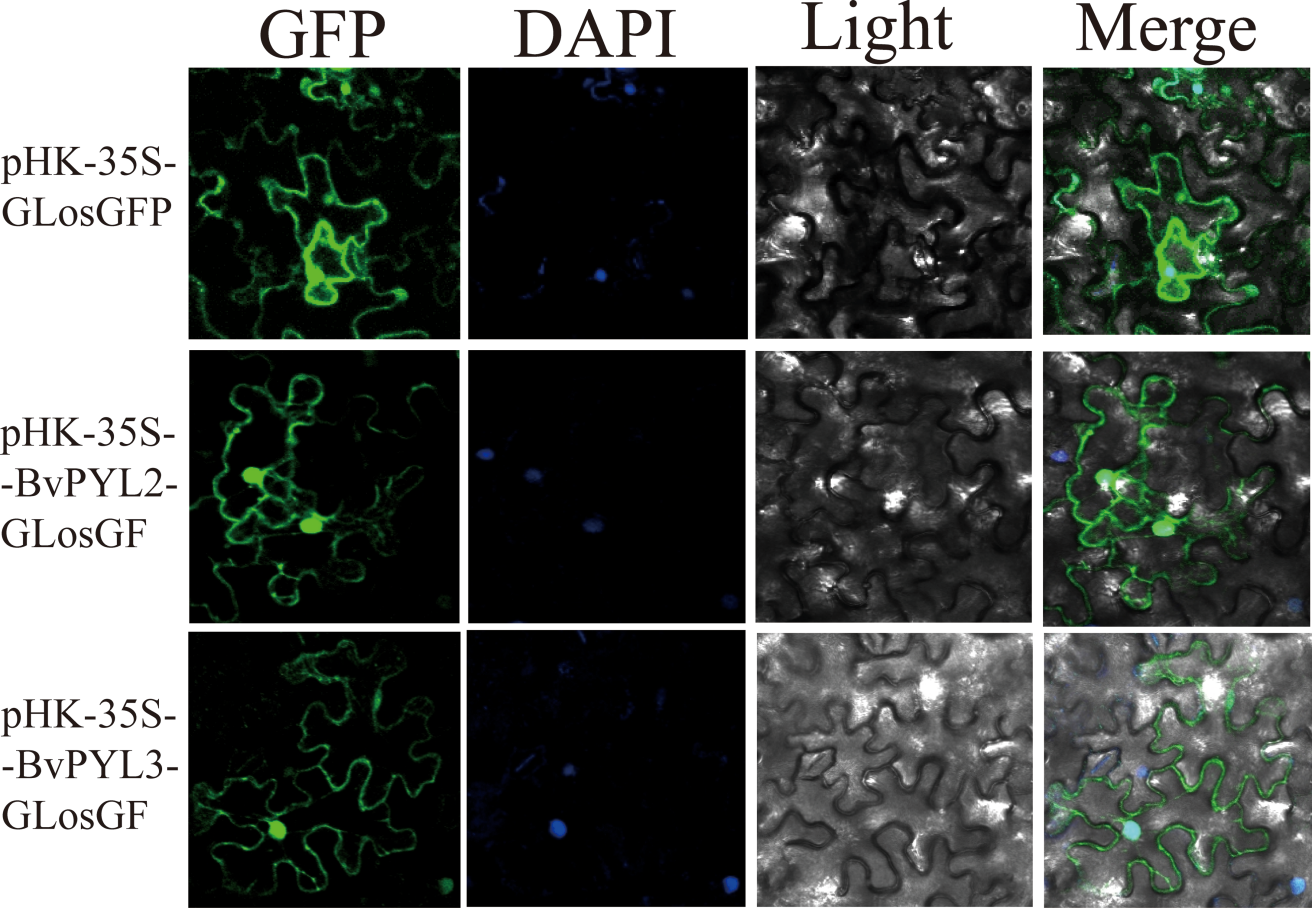
Subcellular localization of *BvPYL2* and *BvPYL3* protein. All fluorescence signals were detected using the confocal microscope. The pHK-35S-GLOSGFP empty plasmid was used as the control. The scale bar was 50 μm.

Transcriptional profiling identified *BvPYL2* and *BvPYL3* as highly expressed genes during *Beta vulgaris*-*Cercospora beticola* interactions. To investigate their subcellular localization, C-terminal GFP fusion constructs (pHK-35S-*BvPYL2*-GLosGFP and pHK-35S-*BvPYL3*-GLosGFP) driven by the cauliflower mosaic virus 35S promoter were generated. These constructs were transformed into Agrobacterium tumefaciens strain GV3101 and transiently expressed in Nicotiana benthamiana leaves via agroinfiltration. Confocal laser scanning microscopy revealed dual localization of both proteins to the plasma membrane, cytoplasm, and nucleus (**Figure 6**). The tripartite distribution pattern suggests multifunctional roles for these ABA receptors, including: extracellular signal perception at the plasma membrane; cytosolic signal transduction; and nuclear transcriptional regulation. The concurrent presence in membrane and nuclear compartments is particularly significant, as it enables simultaneous perception of external stimuli and regulation of nuclear events. This compartment-spanning localization supports their proposed role as central hubs in coordinating defense responses against *CLS*, potentially facilitating rapid signal transduction and transcriptional reprogramming during pathogen challenge (Aerts et al., 2022).

### 3.7 Yeast two-hybrid analysis of BvPYL2/BvPYL3 interactions with BvPP2C37

**Figure 7:**
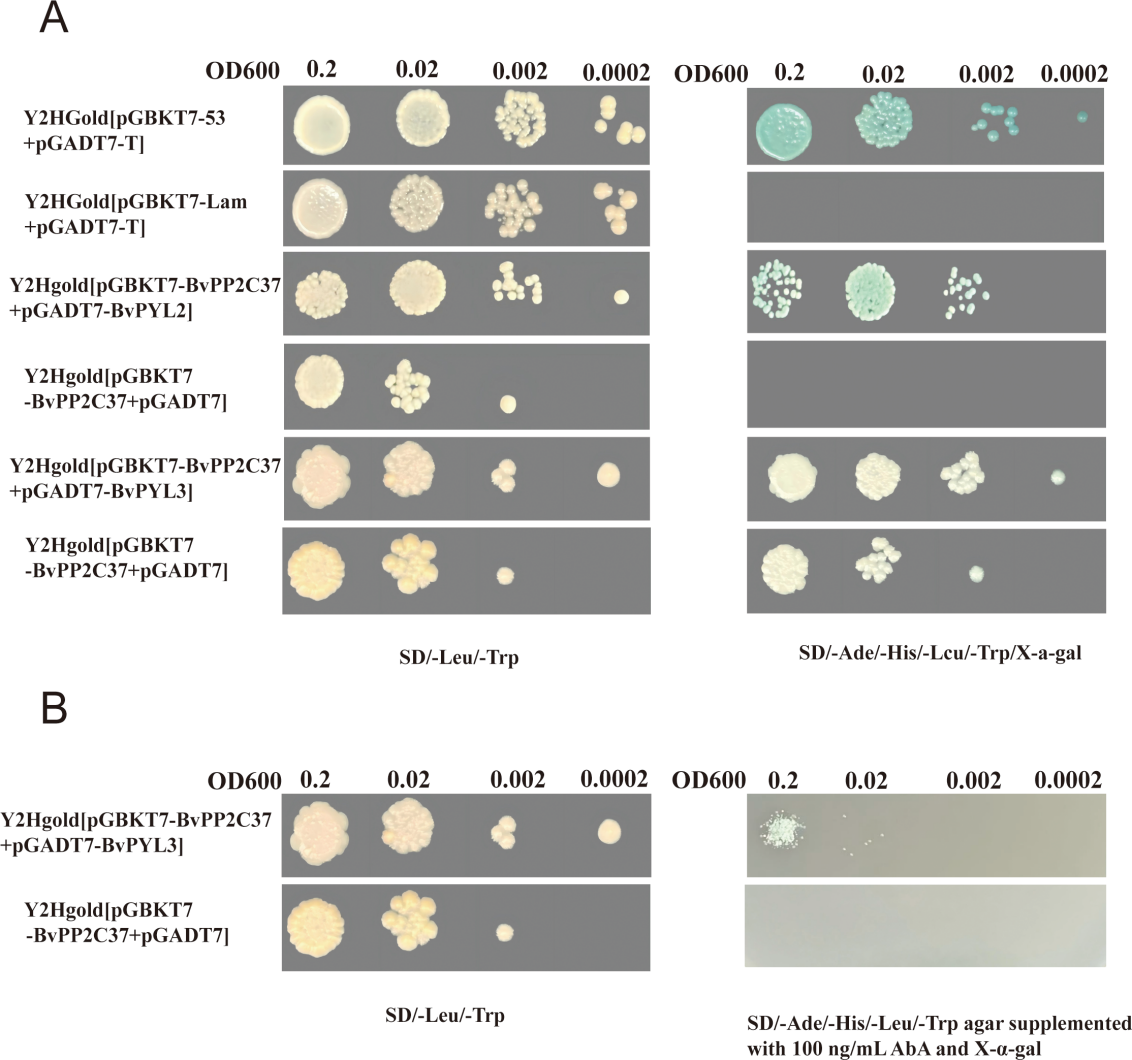
Yeast two-hybrid analysis of interactions between *BvPP2C37* and *BvPYL2/BvPYL3*. **(A)** Serial dilutions (OD600 0.2, 0.02, 0.002, 0.0002) of yeast transformants were spotted on different selection media. Y2HGold [pGBKT7-53 + pGADT7-T] served as the positive control, Y2HGold [pGBKT7-Lam + pGADT7-T] as the negative control, Y2HGold [pGBKT7-*PP2C*37 + pGADT7-*PYL2*] as experimental group 1, Y2HGold [pGBKT7-*BvPP2C37* + pGADT7] as control group 1, Y2HGold [pGBKT7-*PP2C37* + pGADT7-*PYL3*] as experimental group 2, and Y2HGold [pGBKT7-*BvPP2C37* + pGADT7] as control group 2. The interactions were tested on SD/-Leu/-Trp medium and SD/-Ade/-His/-Leu/-Trp/X-α-Gal medium. **(B)** Validation of *BvPYL3- BvPP2C37* interaction under optimized conditions. Y2HGold [pGBKT7-*PP2C*37 + pGADT7-*PYL3*] (experimental group) and Y2HGold [pGBKT7-*BvPP2C37* + pGADT7] (control group) were grown on SD/-Leu/-Trp medium and SD/-Ade/-His/-Leu/-Trp agar supplemented with 100 ng/mL AbA and X-α-Gal. Serial dilutions (OD600 0.2, 0.02, 0.002, 0.0002) were spotted for each condition.

Initial screening of *PP2C* genes was performed using RNA-seq data obtained from *Cercospora beticola*-infected sugar beet (*Beta vulgaris*) leaf tissues. Heatmap analysis (**Supplementary Material**: **Supplementary Figure 3A**) revealed distinct expression patterns among *PP2C* family members. Five genes, including *BVRB_2g025410* and *BVRB_8g197440*, displayed decreasing expression during resistant phases but increasing expression during susceptible phases, while two others maintained stable expression levels. Subsequent qRT-PCR validation confirmed these expression trends (**Supplementary Material**: **Supplementary Figure 3B**). Based on its sustained high expression during resistant stages (R_1_-R_3_) and late-stage upregulation in susceptible conditions (S_4_), *BVRB_2g025410* was selected for further study and designated as *BvPP2C37* due to its homology with *Arabidopsis* orthologs. The potential interaction between *BvPYL2/BvPYL3* and *BvPP2C37* was investigated using a yeast two-hybrid (Y2H) assay. In this system, *BvPP2C37* was fused to the DNA-binding domain (bait vector pGBKT7), while *BvPYL2* and *BvPYL3* were separately fused to the activation domain (prey vector pGADT7). Physical interaction between these proteins would reconstitute a functional transcription factor, activating reporter gene expression. Initial experiments confirmed a specific interaction between *BvPYL2* and *BvPP2C37* (**Figure 7A**). However, testing the *BvPYL3-BvPP2C37* interaction revealed autoactivation (**Figure 7A**), as evidenced by growth on selective medium (SD/-Ade/-His/-Leu/-Trp/X-α-gal) even in the negative control (pGBKT7-*BvPP2C37* + pGADT7). This indicated nonspecific activation of the HIS3 and ADE2 reporter genes independent of protein-protein interaction.

To address the autoactivation issue, the medium was supplemented with aureobasidin A (AbA), a cyclic depsipeptide antibiotic that inhibits fungal IPC synthase (encoded by AUR1). AbA treatment selectively eliminates yeast cells exhibiting background activation while preserving those with genuine protein interactions. After testing a concentration gradient (100–200 ng/mL), 100 ng/mL AbA was determined to be optimal for suppressing autoactivation without compromising valid interactions. Under these optimized conditions (SD/-Ade/-His/-Leu/-Trp agar containing 100 ng/mL AbA and X-α-gal), yeast colonies co-expressing *BvPP2C37* and *BvPYL3* exhibited both growth and α-galactosidase activity (blue coloration; **Figure 7B**), confirming an ABA-dependent interaction (Watanabe et al., 2022). The yeast two-hybrid results demonstrate that both *BvPYL2* and *BvPYL3* can interact with *BvPP2C37*, supporting the formation of a *BvPYL-ABA-BvPP2C37* ternary complex. These findings provide experimental evidence for the role of these interactions in ABA signal transduction during *CLS* infection.

### 3.8 Quantitative analysis of ABA accumulation dynamics and their association with disease resistance in sugar beet

**Figure 8:**
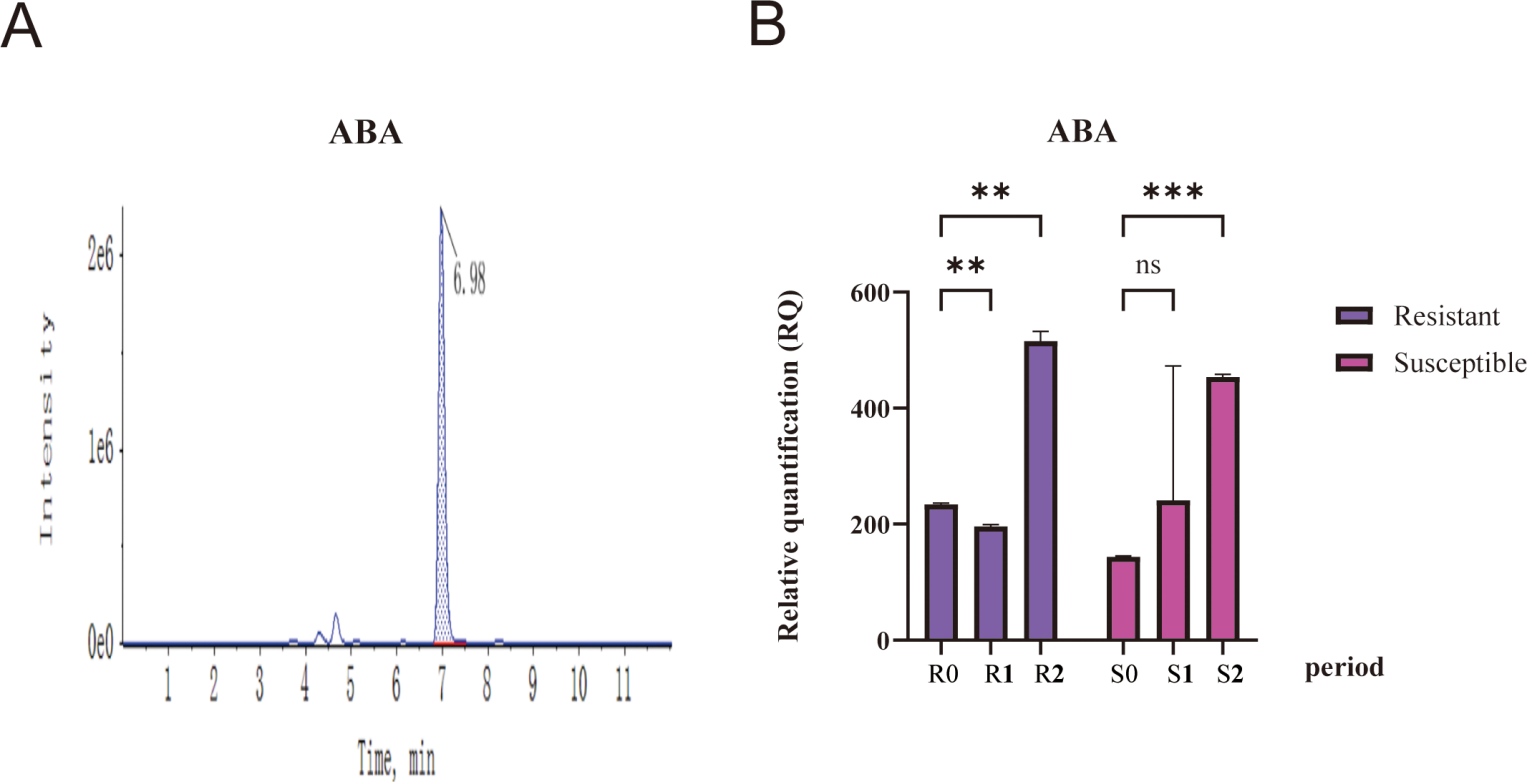
Temporal dynamics of ABA accumulation in sugar beet during *Cercospora beticola* infection. **(A)** LC-MS/MS chromatogram of ABA. **(B)** ABA levels in resistant (81GM241, purple) and susceptible (KWS6661, pink) sugar beet genotype were measured at time points defined from the three-pair true leaf stage: 12 days (0 dpi), 24 days (12 dpi), and 48 days (36 dpi). The data are presented as Relative Quantification (RQ), normalized to the mean value of the resistant genotype at 0 dpi. R0, R1, and R2 correspond to the resistant genotype at 0, 12, and 36 dpi, respectively; S0, S1, and S2 represent the susceptible genotype at the same corresponding time points. Data are expressed as mean relative quantification (RQ) ± standard deviation (SD). Statistical comparisons were conducted using two-way ANOVA with Tukey’s *post-hoc* test (*p < 0.01, **p < 0.001; ns, not significant).

Abscisic acid (ABA) serves as a crucial phytohormone regulating plant defense mechanisms against pathogen invasion, with its temporal accumulation patterns directly influencing the activation of resistance-related signaling pathways. To elucidate the relationship between ABA dynamics and disease resistance phenotypes, endogenous ABA levels were systematically quantified in *Cercospora leaf* sp*ot* (*CLS*)-resistant (81GM241) and susceptible (KWS6661) sugar beet materials throughout progressive infection stages using high-performance liquid chromatography-tandem mass spectrometry (HPLC-MS/MS) (Supplementary Material: **Supplementary Table S8**). Method validation confirmed the specificity of ABA detection, with chromatographic analysis revealing a distinct peak at 0.98 min (intensity: 6.98×10^6^) that precisely matched the retention time and spectral characteristics of authentic ABA standards (**Figure 8A**). This rigorous validation ensured the accuracy of ABA quantification in complex plant matrices.

ABA levels were measured in sugar beet materials at time points defined from the three-pair true leaf stage: 12 days (0 dpi), 24 days (12 dpi), and 48 days (36 dpi), and significant genotypic differences in ABA accumulation patterns were uncovered. The resistant materials exhibited a stage - specific response: maintaining relatively stable baseline ABA levels during the early (R0) and transition (R1) periods. A pronounced accumulation peak emerged at the advanced infection stage (R2), reflecting an intensified ABA - mediated defense response. In contrast, the susceptible materials showed distinct dynamics. It displayed lower ABA levels during the initial (S0) and early - mid (S1) infection phases, with a relatively moderate increase only at the later stage (S2) (**Figure 8B**). Relative quantification analysis corroborated these findings. For resistant sugar beet plants, a remarkable elevation in ABA levels was observed at the R2 stage, which was significantly higher compared to their levels at the R0 and R1 stages. In contrast, for susceptible plants, ABA levels were relatively low at the S0 stage and only showed a moderate increase at the S2 stage. When comparing resistant and susceptible plants at key stages, the resistant group at R2 had significantly higher ABA levels than the susceptible group at most stages, while no significant difference was found between S1 and S2 of the susceptible group (ns). Collectively, these results illustrate that *Cercospora beticola* infection specifically triggers differential ABA accumulation patterns, with resistant materials exhibiting a more pronounced and stage specific ABA elevation that likely contributes to defense responses.

The ABA accumulation profiles showed remarkable temporal coordination with the expression patterns of key ABA signaling components. As previously established through qRT-PCR analysis, the ABA receptors *BvPYL2* and *BvPYL3* exhibited maximal transcriptional induction in resistant plants precisely during the R_2_ stage, coinciding with peak ABA accumulation. This temporal coupling suggests that sustained ABA responses in resistant genotypes are amplified through enhanced receptor-mediated signal transduction. Conversely, the attenuated ABA response in susceptible plants may reflect compromised signaling capacity, potentially due to the prolonged upregulation of *BvPP2C37*—a negative regulator of ABA signaling that was shown through yeast two-hybrid assays to potentially inhibit downstream *SnRK2* kinase activity via dephosphorylation. These findings establish that both the magnitude and temporal dynamics of ABA accumulation constitute critical determinants of *CLS* resistance in sugar beet. The resistant genotype employs a delayed but potent ABA surge coupled with coordinated receptor upregulation to mount effective defenses, while the susceptible genotype fails to maintain this response, likely due to *PP2C*-mediated suppression of ABA signaling. This ABA-centric regulatory module provides a mechanistic basis for understanding genotype-specific resistance to *CLS* infection, offering potential targets for molecular breeding strategies.

### 3.9 ABA-mediated stomatal defense against *Cercospora beticola* infection

**Figure 9:**
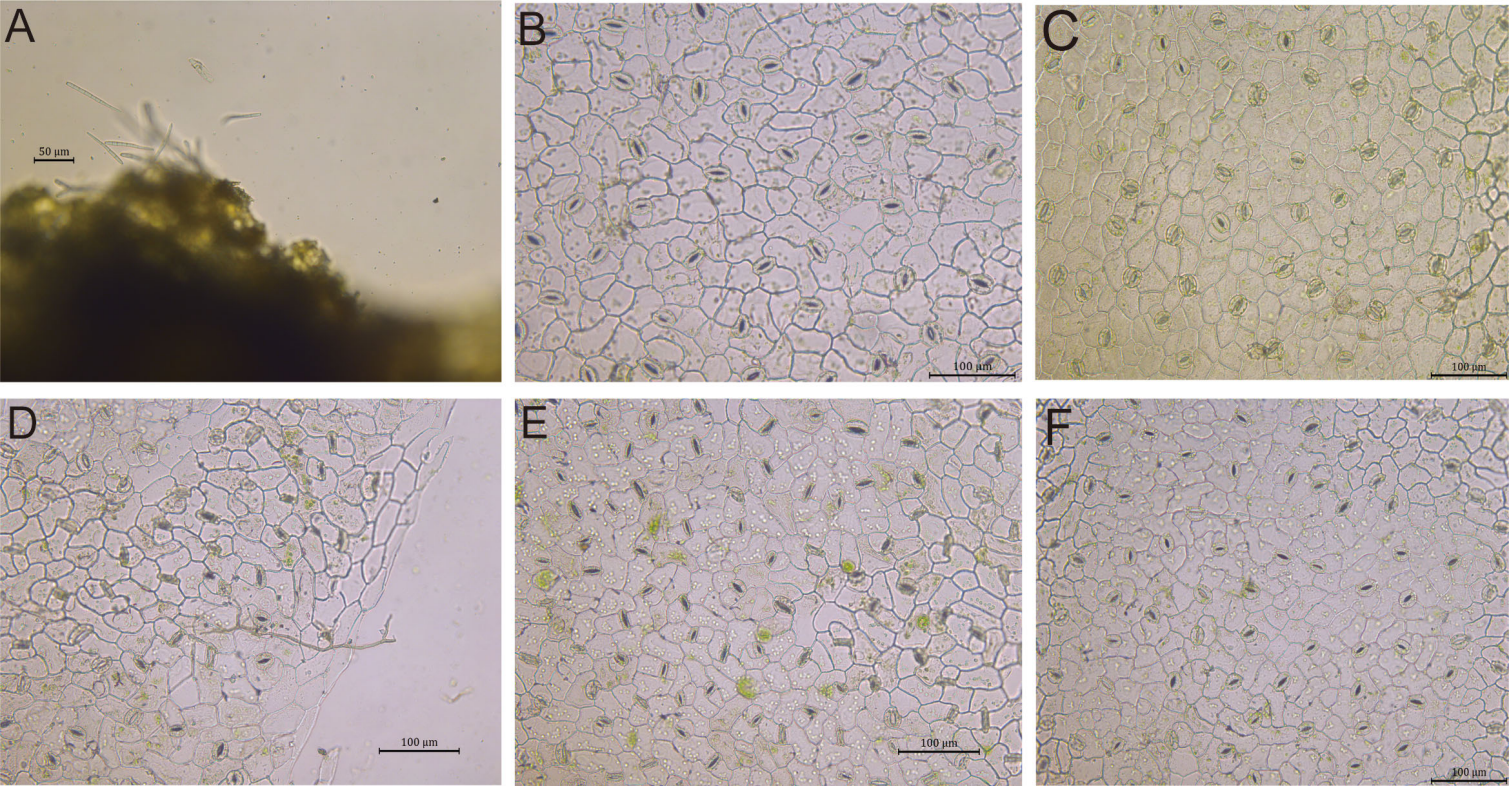
ABA-mediated stomatal defense in sugar beet leaves against *Cercospora beticola* infection. **(A)***CLS* conidia on leaf stomata (50 µm scale bar). **(B)** Open stomata with turgid guard cells in healthy leaves. **(C)** Guard cell detail of healthy leaves (100 µm scale bar). **(D)** Partial stomatal closure at early infection. **(E)** Complete stomatal closure with guard cell collapse (effective ABA signaling). **(F)** Persistent stomatal opening and hyphal penetration (compromised defense; 100 µm scale bar).

The ascomycete fungus *Cercospora beticola (Sacc.)*, the etiological agent of *Cercospora leaf* sp*ot* (*CLS*) in sugar beet (*Beta vulgaris L.*), has evolved specialized pathogenic adaptations for host colonization. Its infection cycle is initiated through stomatal penetration, a process facilitated by the production of needle-shaped conidia that exhibit morphological specialization for efficient stomatal entry. This infection strategy represents a key virulence determinant in the pathogen’s life cycle. (**Figure 9A**). Under non-stress conditions, sugar beet leaves maintain fully open stomata characterized by turgid guard cells (**Figures 9B, C**). The ABA-mediated defense response unfolds through three mechanistically distinct phases: an initial response phase featuring partial guard cell contraction coupled with ABA signaling pathway activation (**Figure 9D**); an effective defense phase marked by complete stomatal closure through guard cell collapse, mediated by the canonical *PYL-PP2C-SnRK2* signaling module and subsequent regulation of ion channels (**Figure 9E**); and a compromised defense phase where persistently open stomata facilitate hyphal penetration, indicative of disrupted ABA signal transduction (**Figure 9F**). This coordinated physiological response involves sequential molecular events including receptor activation, ion flux coordination, and osmotic potential adjustment, culminating in stomatal closure (Chen K. et al., 2020). All morphological observations were systematically validated through comprehensive microscopic examination.

## 4 Discussion

The *PYR/PYL/RCAR (PYL)* gene family constitutes a central regulatory component of abscisic acid (ABA) signaling pathways and plays a pivotal role in modulating plant responses to environmental stress (Fujii et al., 2009; Park et al., 2009). Although these receptors have been extensively investigated in model organisms such as *Arabidopsis thaliana* and major crops like *Oryza sativa* (Zhang et al., 2015; Yadav et al., 2020), their functional roles in sugar beet (*Beta vulgaris L*.) remain largely undefined.

Evolutionary Conservation and Functional Diversification of *BvPYL* Genes;Phylogenetic analysis of the sugar beet genome identified ten *BvPYL* genes that are distributed across four well-supported clades, indicating substantial evolutionary conservation with their orthologs in both *Arabidopsis thaliana* and *Oryza sativa*. Notably, *BvPYL2* and *BvPYL4* cluster specifically with the *AtPYL7–10* subgroup, which has been functionally characterized as regulators of stomatal immunity and pathogen defense responses under diverse abiotic stress conditions (Chen et al., 2017; Sun et al., 2017; García-León et al., 2019). This phylogenetic conservation strongly suggests that *BvPYL2* and *BvPYL4* have retained similar dual roles in mediating both biotic and abiotic stress responses in sugar beet. The evolutionary trajectory of *PYL* genes appears to follow consistent patterns across plant species. Comparative analyses have demonstrated that paralogous *PYL* genes in pomegranate (*PgPYLs*) and strawberry (*FaPYL3/4*) have undergone similar subfunctionalization processes, acquiring specialized functions in drought tolerance and fruit ripening regulation, respectively (Yin et al., 2024; Jia et al., 2025). Importantly, the identification of a segmental duplication event involving *BvPYL2* and *BvPYL4* provides direct evidence that gene duplication serves as a key mechanism driving functional diversification within the *PYL* gene family. This conclusion is further corroborated by comparable findings in *Arabidopsis thaliana*, *Oryza sativa*, and Gossypium hirsutum, indicating that this evolutionary mechanism is broadly conserved among angiosperms (Zhang et al., 2017; Lan et al., 2024).

Mechanistic Divergence in *BvPYL2* and *BvPYL3*-Mediated ABA Signaling Pathways;Systematic investigations have delineated two functionally distinct mechanisms through which *BvPYL2* and *BvPYL3* regulate ABA signaling cascades. Biochemical analyses demonstrate that *BvPYL2* establishes constitutive interactions with the protein phosphatase *BvPP2C37* in an ABA-independent manner, a regulatory pattern that parallels the *BnaPYL1/9*-mediated suppression of *BnaPP2C37* activity in *Brassica napus*, which enhances drought tolerance through direct phosphatase inhibition (Zhai et al., 2024). In contrast, *BvPYL3* exhibits strict ABA-dependence for stable complex formation with *BvPP2C37*, mirroring the ABA-dependent activation of *MsPYL6/9* in *Medicago sativa* that induces stomatal closure and reduces transpiration rates under drought conditions (Zhao et al., 2025). This functional dichotomy between receptor isoforms represents an evolutionarily conserved strategy for optimizing stress response regulation. *BvPYL2* maintains basal signaling through its ABA-independent engagement with *PP2C* phosphatases, whereas *BvPYL3* provides precise hormonal modulation of signaling pathways. The conservation of this regulatory specialization is evidenced by: the ABA-dependent interaction between *BSPP2C22* and *BSPYL* in *Bletilla striata*, which coordinates responses to multiple abiotic stresses (Liu S. et al., 2022); and the *BnaPYL1/9*-mediated negative regulation of *BnaPP2C37* in *Brassica napus*, establishing a feedback loop with ABF transcription factors to fine-tune drought responses (Zhai et al., 2024) Signaling transduction of ABA, ROS, and Ca2+ in plant stomatal closure in response to drought. These findings establish that differential ABA responsiveness among *PYL* isoforms constitutes a fundamental adaptive mechanism enabling plants to precisely modulate their physiological responses to diverse environmental challenges. The coexistence of both ABA-dependent and -independent regulatory modules provides plants with the flexibility to maintain basal stress signaling while allowing for hormone-responsive modulation of defense mechanisms.

Molecular Mechanisms of *PYL-PP2C*-Mediated Stomatal Defense Against Pathogen Invasion;The *PYL-PP2C* module serves as a core regulatory hub in stomatal immunity, integrating abscisic acid (ABA) signaling with pathogen defense responses (Zeng et al., 2020; Shi et al., 2022). Upon pathogen perception, this system orchestrates rapid guard cell signaling reprogramming, involving phosphorylation-dependent modulation of ABA, salicylic acid, calcium, and reactive oxygen species (ROS) cascades, as evidenced by proteomic analyses (Pang et al., 2020). Concurrently, spatiotemporally controlled fluctuations in secondary messengers like ROS and Ca²^+^ drive stomatal closure, forming a physical barrier against pathogen entry (Liu H. et al., 2022).The system’s adaptability stems from isoform-specific *PYL-PP2C* interactions and crosstalk with brassinosteroid and drought stress pathways (Chen et al., 2024; Pri-Tal et al., 2024). For instance, *Arabidopsis* RAF-like kinases enhance subclass III *SnRK2* activity under osmotic stress (Komatsu et al., 2020), while wheat *TaPYL4* improves drought adaptation via osmotic regulation. Notably, the protein stability of *TaPYL4* may be further modulated by E3 ubiquitin ligases such as *TaPUB1* (Zhang et al., 2021). Feedback mechanisms, such as *BvPYL3* in sugar beet (*Beta vulgaris*) has identified these ABA receptors as key regulators of defense responses against *Cercospora beticola*, offering promising targets for molecular breeding. The subcellular distribution patterns of *BvPYL2* and *BvPYL3*, while providing valuable preliminary insights into their functional complexity, require further validation using more precise localization techniques. The current observations based on 35S promoter-driven expression suggest potential localization at multiple cellular sites, but definitive distinction between plasma membrane, cytosolic, and nuclear compartments will require implementation of specialized approaches such as plasmolysis assays and co-localization studies with specific organelle markers. These technical considerations notwithstanding, the differential ABA sensitivity exhibited by *BvPYL2* (constitutive activity) and *BvPYL3* (ABA-dependent activation), combined with their distinct subcellular distribution trends, establishes a preliminary framework for understanding their roles in ABA signaling and provides a basis for developing targeted crop improvement strategies. These findings suggest three concrete approaches for genetic enhancement: precise modulation of receptor expression through tissue-specific or stress-inducible promoters; optimization of receptor-phosphatase interactions via engineering of key *PP2C*-binding domains; and utilization of conserved cis-regulatory elements to develop ABA-responsive expression systems (Ortigosa et al., 2019). To facilitate the translation of these molecular insights into practical breeding applications, several strategies can be employed to achieve targeted upregulation of *BvPYL* genes. These include the use of tissue-specific or pathogen-responsive promoters to drive expression in stomatal guard cells or leaf tissues, CRISPR/Cas9-mediated genome editing to modify promoter regions or create gain-of-function alleles, and marker-assisted selection to pyramid favorable *BvPYL* haplotypes in elite sugar beet varieties. The natural variation in ABA-sensitivity between these receptor isoforms serves as a valuable template for designing customized response thresholds to pathogen challenge, potentially enabling the development of sugar beet varieties with enhanced innate immunity while maintaining optimal growth and yield characteristics under normal conditions.

## 5 Conclusions

This study systematically characterized the *BvPYL* gene family in sugar beet (*Beta vulgaris*), constructing a model of *BvPYL*-mediated responses to *Cercospora beticola* infection. Genome-wide identification revealed 10 members of the *BvPYL* gene family in sugar beet. Genomic, bioinformatic, and expression analyses indicated that *BvPYL2* and *BvPYL3* are significantly regulated by *Cercospora beticola* infection, suggesting that *BvPYL*s play a crucial role in sugar beet’s defense against this pathogen. Moreover, the coordinated action of *BvPYL2* and *BvPYL3*, coupled with ABA accumulation, facilitates stomatal closure to restrict fungal entry. Additionally, protein interaction studies uncovered divergent regulatory mechanisms within the *BvPYL* family: *BvPYL2* constitutively interacts with *BvPP2C37*, while *BvPYL3* forms an ABA-dependent complex. This study sheds light on the potential roles of ABA receptors in plant defense responses against fungal pathogens, providing a deeper understanding of the ABA signaling pathway and its interaction with biotic stresses in sugar beet.

## Data Availability

The datasets presented in this study can be found in online repositories. The names of the repository/repositories and accession number(s) can be found in the article/**Supplementary Material**.

## Supplementary material

Supplementary Figure 1 Distribution of *BvPYL* family genes on the chromosomes of *Beta vulgari.*

Supplementary Figure 2 The multiple sequence alignment results of the *BvPYL* protein.

Supplementary Figure 3 Expression Analysis of *BvPP2C* Genes in Response to *Cercospora beticola* Infection.

Supplementary Table 1 Primers used in this study.

Supplementary Table 2 Sugarbeet *PYL-PP2C-SnRK2s* gene group.

Supplementary Table 3 KEGG enrichment analysis statistics.

Supplementary Table 4 GO enrichment analysis statistics.

Supplementary Table 5 Physicochemical properties of the 10 *BvPYL* gene family members.

Supplementary Table 6 Protein sequences of the plant *PYLs.*

Supplementary Table 7 *BvPYL* transcriptome data.

Supplementary Table 8 Detection results of plant hormones ABA in 18 beet leaves.
