# Supplementary material for: Molecular profiling of PYL gene family in sugar beet (Beta vulgaris L.) and BvPYL2/3 involved in ABA accumulation confers enhanced resistance to CLS (Cercospora Leaf Spot)
Source: Front Plant Sci. 2025 Dec 4;16:1694558. doi: 10.3389/fpls.2025.1694558 (PMC12711715; doi:10.3389/fpls.2025.1694558)
Supplement: Supplementary Figure 1 — Distribution of BvPYL family genes on the chromosomes of Beta vulgari. [file DataSheet1.zip › Supplementary Materials/Table S1.docx]

Table S1. Primers used in this study

| **Primer name** | **primer sequence (5'→3')** | **Purpose** |
| --- | --- | --- |
| *BvACTIN-F* | ACTGGTATTGTGCTTGACTC | qPCR |
| *BvACTIN-R* | ATGAGATAATCAGTGAGATC | qPCR |
| *BvPYL1-qPCR-F* | GCCTAACCAAAGAGGAGTAC | qPCR |
| *BvPYL1-qPCR-R* | TCATTCATTTCCGTGCATTG | qPCR |
| *BvPYL2-qPCR-F* | AGACACCACAATCACAAGAT | qPCR |
| *BvPYL2-qPCR-R* | GTAGCACGTCTCCTCTTTAG | qPCR |
| *BvPYL3-qPCR-F* | ACTCACACCACAACAAGTAT | qPCR |
| *BvPYL3-qPCR-R*  *BvPYL4-qPCR-F*  *BvPYL4-qPCR-R*  *BvPYL5-qPCR-F*  *BvPYL5-qPCR-R*  *BvPYL6-qPCR-F*  *BvPYL6-qPCR-R*  *BvPYL7-qPCR-F*  *BvPYL7-qPCR-R*  *BvPYL8-qPCR-F*  *BvPYL8-qPCR-R*  *BvPYL9-qPCR-F*  *BvPYL9-qPCR-R*  *BvPYL10-qPCR-F*  *BvPYL10-qPCR-R*  *BVRB_7g173230 -qPCR-F*  *BVRB_7g173230 -qPCR-R*  *BVRB_5g098880 -qPCR-F*  *BVRB_5g098880 -qPCR-R*  *BVRB_3g052290-qPCR-F*  *BVRB_3g052290 -qPCR-R*  *BVRB_8g199360 -qPCR-F*  *BVRB_8g199360 -qPCR-R*  *BVRB_8g197440 -qPCR-F*  *BVRB_8g197440 -qPCR-R*  *BVRB_2g025410 -qPCR-F*  *BVRB_2g025410 -qPCR-R*  *BVRB_2g039100 -qPCR-F*  *BVRB_2g039100 -qPCR-R*  *BvPYL2F*  *BvPYL2R*  *BvPYL3F*  *BvPYL3R*  *BvPYL2F*  *BvPYL2R*  *BvPYL3F*  *BvPYL3R*  *BvPP2C37F*  *BvPP2C37R* | CCATGGCTTGAGCAATACC  GGATATGGCGGAGTTGAAG  TTGTACAGCCAAACGTTCT  CATTCTATGTCCCAGACGAC  TCGTCTAGCTAAATTCTCAGC  TTCGCATCCCTAATACCAAG  TCACCTGTTTTCCATGGC  TGAGGTAGATGTGGAAGTAAAG  TCATCAAGGTCCATGAAAGT  ACTTCCCAAAGTCTTACAGC  AGGCGTTCATCTCCGATAT  ACCATAAGCAACTGGATGTT  AACAAGACTGGTCTGTTCAG  CCCTCGTTAGCTCATGAATT  CAGCGATCTTTGTAAGGGAT  AACTTCCTCTACACAAGCTG  GTGAAATCTATCTCGGCACT  TGAATGAATGGGAGAGTGTG  TAGCTCAACCAACATAGCAG  CGCATAAATCGAGGACTACA  ATTCTAGCCAATTCGTCTGG  AGCTTTGAACCTGTGAGTAG  TAGCTTCAATTTCCTCCACC  CTGAGGTTGCTGACTATTGT  ATGACATCCCAAAGTCCATC  TTACGTCTGTTTGTGGTAGG  CTCTTGCACCATCCCAATAT  CCTTGTGATCAACAGTTAAAGG  GCAGAACAAGTAGAAAGAAGC  AACACGGGGGACTTTGCAACATGGAGTCTGTATACATTTCTAG  CCTGAAGCGGCCGCTGTACATGCATTTGCCATTCTCTC  AACACGGGGGACTTTGCAACATGGGAGATCAAGAATCAAG  CCTGAAGCGGCCGCTGTACAACTTTGATCAGAAAAAGCTTC  GccatggaggccagtgaattcATGGAGTCTGTATACATTTCTAGACACCA  CagctcgagctcgatggatccTCATGCATTTGCCATTCTCTCA  GccatggaggccagtgaattcATGGGAGATCAAGAATCAAGCAA  CagctcgagctcgatggatccTTAACTTTGATCAGAAAAAGCTTCTTC  AtggccatggaggccgaattcATGGCGGGAATGTGCTGTGGAATTAAAG  ccgctgcaggtcgacggatccTCATCTCCTAAGATCAACTACAACAAC | qPCR  qPCR  qPCR  qPCR  qPCR  qPCR  qPCR  qPCR  qPCR  qPCR  qPCR  qPCR  qPCR  qPCR  qPCR  qPCR  qPCR  qPCR  qPCR  qPCR  qPCR  qPCR  qPCR  qPCR  qPCR  qPCR  qPCR  qPCR  Qpcr  subcellular localization  subcellular localization  subcellular localization  subcellular localization  Y2H  Y2H  Y2H  Y2H  Y2H  Y2H |

Notes: F and R represent forward (sense) and reverse (anti-sense) primers, respectively.
