# Supplementary material for: Molecular profiling of PYL gene family in sugar beet (Beta vulgaris L.) and BvPYL2/3 involved in ABA accumulation confers enhanced resistance to CLS (Cercospora Leaf Spot)
Source: Front Plant Sci. 2025 Dec 4;16:1694558. doi: 10.3389/fpls.2025.1694558 (PMC12711715; doi:10.3389/fpls.2025.1694558)
Supplement: Supplementary Figure 1 — Distribution of BvPYL family genes on the chromosomes of Beta vulgari. [file DataSheet1.zip › Supplementary Materials/Table S5.docx]

**Table S5.**

Physicochemical properties of the 10 *Bv*PYL gene family members.

| **Gene Name** | **Gene ID** | **Number of amino**  **acids** | **Molecular weight (Mw/**  **Da)** | **Theoretical （pI)** | **Instability**  **index** | **Aliphatic**  **index** | **Grand average of**  **hydropathicity** | **Subcellular**  **localization**  **prediction** |
| --- | --- | --- | --- | --- | --- | --- | --- | --- |
| *BvPYL1* | *BVRB_006920* | 197 | 22002.69 | 6.05 | 41.04 | 75.58 | -0.513 | Cell membrane/Cytoplasm. |
| *BvPYL2* | *BVRB_1g003320* | 168 | 18806.37 | 6.70 | 44.18 | 97.88 | -0.256 | Nucleus |
| *BvPYL3* | *BVRB_2g032630* | 164 | 18336.08 | 4.47 | 30.88 | 82.07 | -0.029 | Cytoplasm |
| *BvPYL4* | *BVRB_3g052410* | 187 | 21075.99 | 5.90 | 41.84 | 92.09 | -0.324 | Chloroplast |
| *BvPYL5* | *BVRB_5g103280* | 223 | 24986.83 | 6.61 | 51.08 | 84.17 | -0.425 | Cytoplasm |
| *BvPYL6* | *BVRB_5g105930* | 234 | 25617.64 | 5.71 | 32.87 | 79.23 | -0.351 | Nucleus |
| *BvPYL7* | *BVRB_6g149380* | 151 | 17238.78 | 4.92 | 25.20 | 84.64 | -0.281 | Cytoplasm |
| *BvPYL8* | *BVRB_9g211290* | 184 | 20860.75 | 5.82 | 40.96 | 88.91 | -0.322 | Cytoplasm |
| *BvPYL9* | *BVRB_1g019210* | 181 | 20376.85 | 5.10 | 52.09 | 84.48 | -0.406 | Nucleus |
| *BvPYL10* | *BVRB_2g044620* | 261 | 28553.28 | 6.59 | 41.99 | 75.71 | -0.264 | Cytoplasm/Nucleus |
