# Supplementary figures and images for: Molecular profiling of PYL gene family in sugar beet (Beta vulgaris L.) and BvPYL2/3 involved in ABA accumulation confers enhanced resistance to CLS (Cercospora Leaf Spot)
Source: Front Plant Sci. 2025 Dec 4;16:1694558. doi: 10.3389/fpls.2025.1694558 (PMC12711715; doi:10.3389/fpls.2025.1694558)

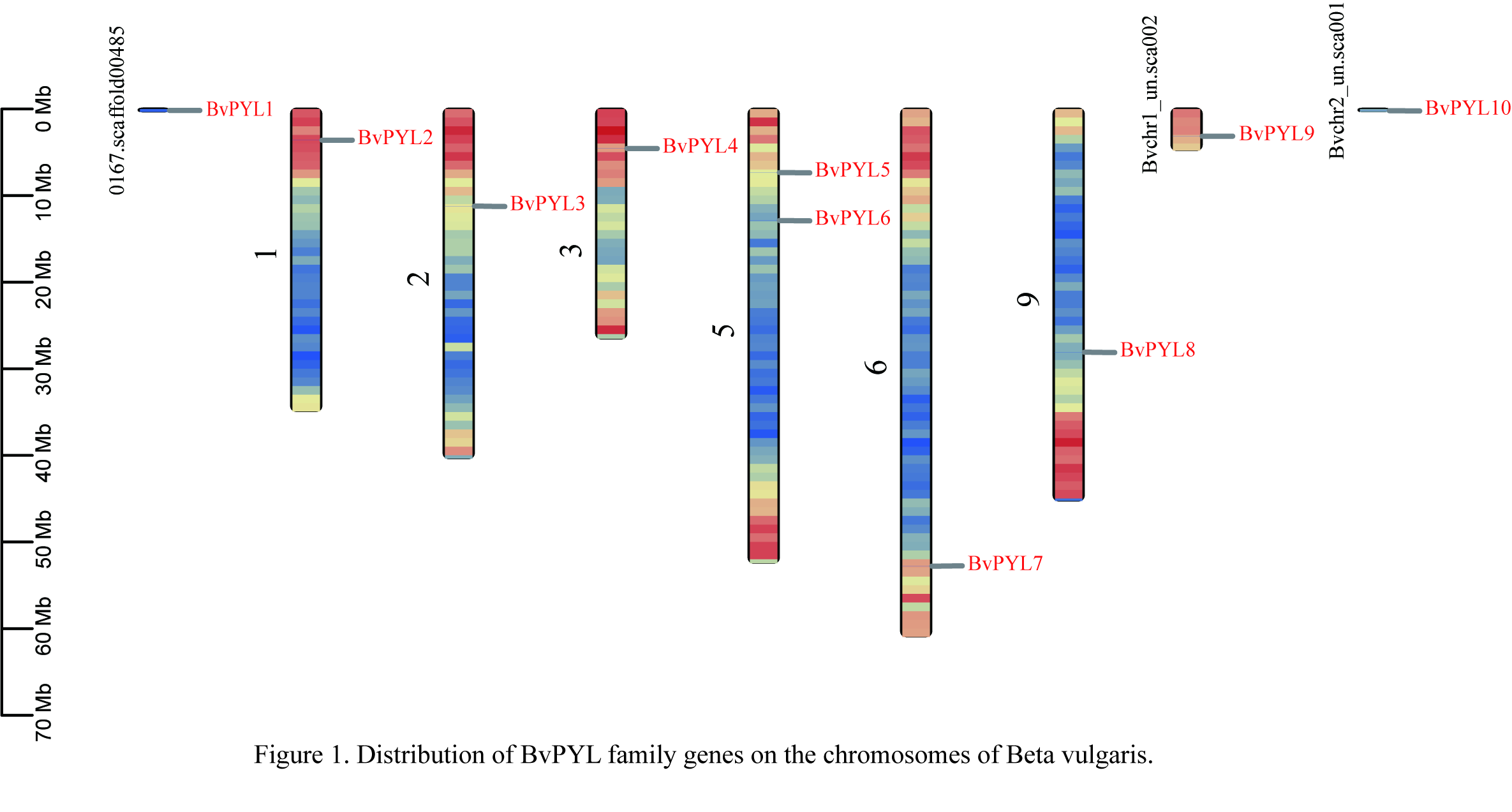

Supplement: Supplementary Figure 1 — Distribution of BvPYL family genes on the chromosomes of Beta vulgari. [file DataSheet1.zip › Supplementary Materials/Figure 1.tif]

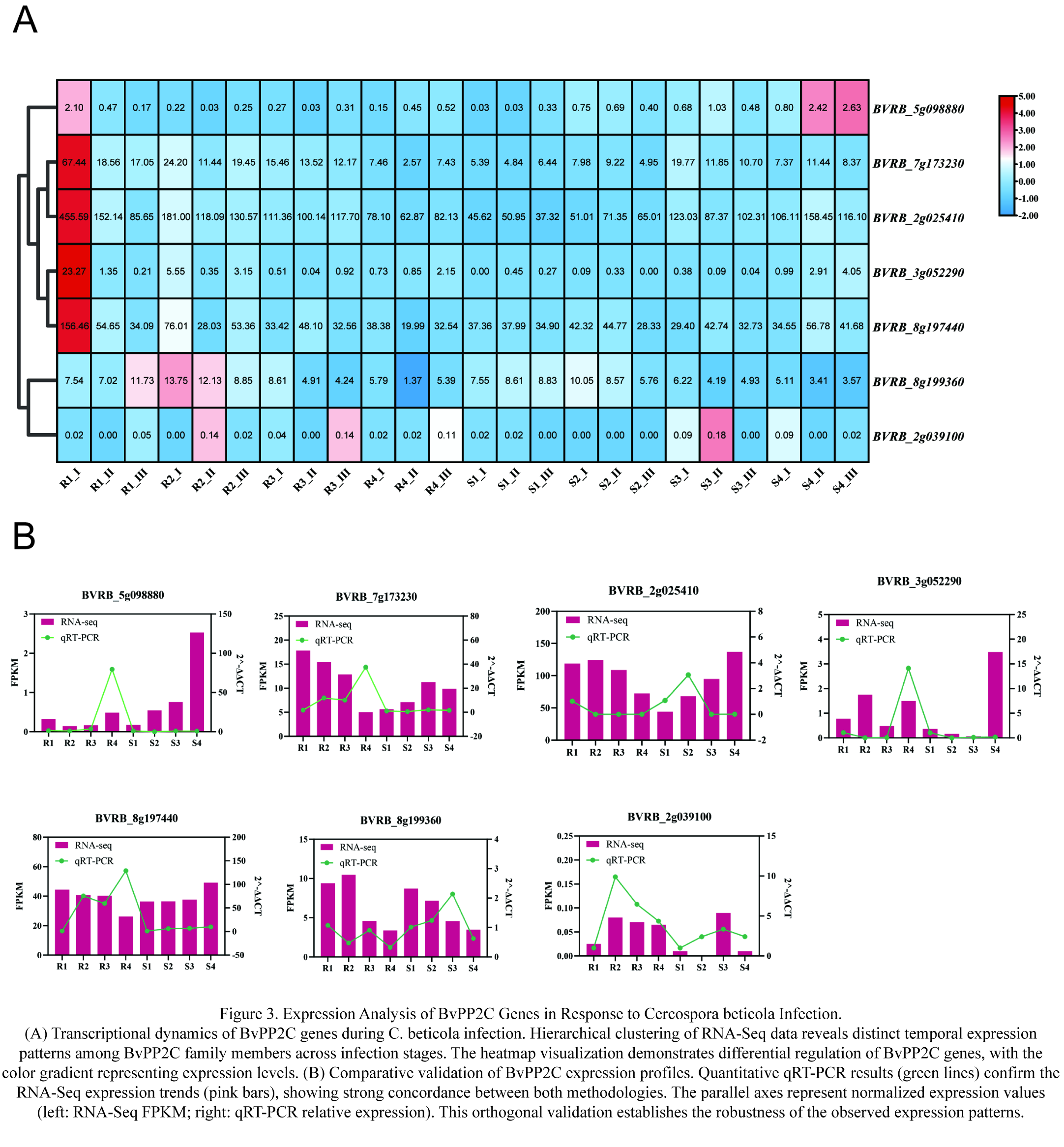

Supplement: Supplementary Figure 1 — Distribution of BvPYL family genes on the chromosomes of Beta vulgari. [file DataSheet1.zip › Supplementary Materials/Figure 3.tif]

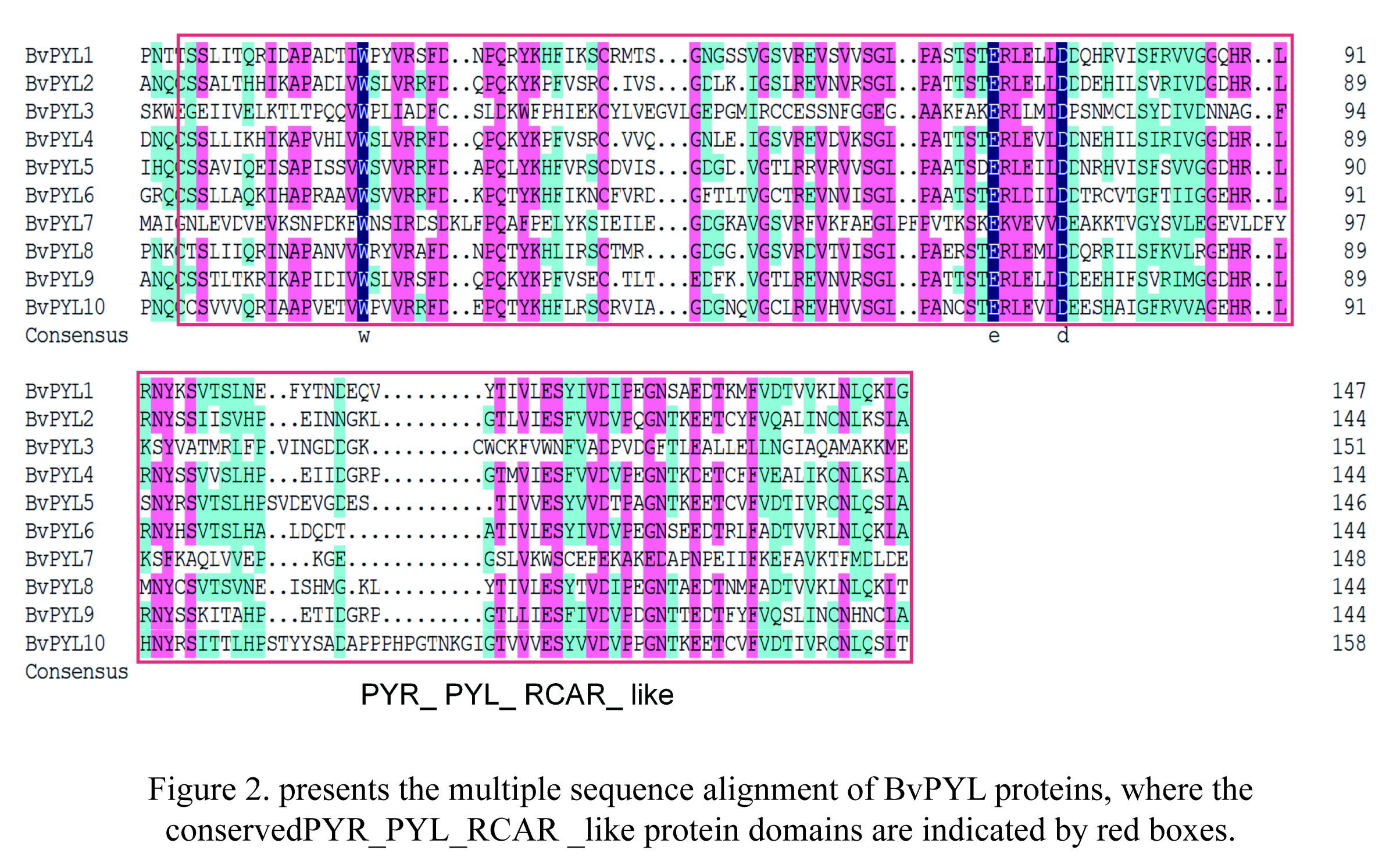

Supplement: Supplementary Figure 1 — Distribution of BvPYL family genes on the chromosomes of Beta vulgari. [file DataSheet1.zip › Supplementary Materials/Figure 2 .tif]
